# CENPF knockdown inhibits adriamycin chemoresistance in triple-negative breast cancer via the Rb-E2F1 axis

**DOI:** 10.1038/s41598-023-28355-z

**Published:** 2023-01-31

**Authors:** Depeng Wang, Wei Xu, Minghua Huang, Wei Ma, Yulu Liu, Xingchen Zhou, Qingrui Yang, Kun Mu

**Affiliations:** 1 Department of Pathology, School of Basic Medical Sciences, Shandong University, Wen Hua Xi Road 44, Jinan, 250012 China; 2 Department of Rheumatology and Immunology, Shandong Provincial Hospital, Shandong University, Jingwu Road 324, Jinan, 250021 China; 3 Pathology Department, First Affiliated Hospital of Weifang Medical University (Weifang People’s Hospital), Weifang, China; 4 Department of Respiratory Medicine, Shandong Provincial Third Hospital, Shandong University, Jinan, 250132 China; 5 Department of Pathology, The Second Hospital of Shandong University, Jinan, 250033 China; 6 Department of Rheumatology and Immunology, Shandong Provincial Hospital Affiliated to Shandong First Medical University, Jinan, 250021 Shandong China; 7 Department of Pathology, Qilu Hospital of Shandong University, Jinan, 250012 China

**Keywords:** Breast cancer, Cancer therapy

## Abstract

Drug resistance occurs frequently in triple-negative breast cancer (TNBC) and leads to early relapse and short survival. Targeting the DNA damage response (DDR) has become an effective strategy for overcoming TNBC chemoresistance. CENPF (centromere protein) is a key regulator of cell cycle progression, but its role in TNBC chemotherapy resistance remains unclear. Here, we found that CENPF, which is highly expressed in TNBC, is associated with a poor prognosis in patients receiving chemotherapy. In addition, in vitro CENPF knockdown significantly increased adriamycin (ADR)-induced cytotoxicity in MDA-MB-231 cells and ADR-resistant cells (MDA-MB-231/ADR). Then, we demonstrated that CENPF targets Chk1-mediated G2/M phase arrest and binds to Rb to compete with E2F1 in TNBC. Considering the crucial role of E2F1 in the DNA damage response and DNA repair, a novel mechanism by which CENPF regulates the Rb-E2F1 axis will provide new horizons to overcome chemotherapy resistance in TNBC.

## Introduction

Breast cancer is the second most common cancer worldwide after lung cancer and the leading cause of cancer-related death in women^1^. TNBC represents 10–15% of breast cancers and is the most lethal subtype due to its high heterogeneity, aggressive nature, and lack of treatment options^2^. Chemotherapy remains the mainstay of treatment for TNBC. Unfortunately, drug resistance occurs frequently in TNBC and is a major cause of early relapse and short survival^3^.

Dysregulation of cell cycle control mechanisms allows TNBC cells to continuously malignant proliferation. DNA replication, a central event for cancer cell proliferation, can be disrupted by anthracyclines by inhibiting topoisomerase II^4^. Exogenous DNA-damaging agents trigger cancer cells to activate DDR pathways, including cell cycle arrest and DNA repair^5^. Obviously, these protective DDRs confer tumour chemoresistance. Targeting DDRs has become an effective strategy for overcoming TNBC chemoresistance^6,7^.

In our previous research on the mechanism of Chk1 in TNBC chemotherapy resistance, we found that CENPF acts as the upstream regulatory molecule of Chk1^8^. CENPF is an evolutionarily highly conserved protein related to kinetochore structure and function. The centromere-kinetochore complex mediates the binding of spindle filaments to chromosomes and is the structural basis for the orderly pairing and segregation of chromosomes^9,10^. CENPF has been found to be overexpressed in various malignant tumours, such as prostate cancer, breast cancer, and nasopharyngeal carcinoma, and to activate signalling pathways related to malignant tumour progression to promote the proliferation and metastasis of cancer cells^11–13^. Recent studies have shown that abnormal expression of CENPF is also closely related to chemotherapy resistance in ovarian cancer and lung adenocarcinoma^14,15^. At present, the mechanism by which CENPF is involved in TNBC chemotherapy resistance has not been elucidated.

Based on a previous study, we further explored the mechanism by which CENPF is involved in TNBC chemotherapy resistance through a series of bioinformatics analyses and molecular biology experiments. Our current study revealed the role of CENPF in TNBC chemotherapy resistance, and CENPF targets Chk1-mediated G2/M phase arrest and binds to Rb to compete with E2F1. Considering the crucial role of E2F1 in the DNA damage response and DNA repair, we hope this study will provide a new intervention target to overcome chemotherapy resistance in TNBC.

## Material and methods

### Bioinformatics analysis

We used the following bioinformatics analysis strategies and tools at each experimental stage. We measured CENPF expression in TNBC and adjacent peritumoral tissues from The Cancer Genome Atlas (TCGA) and the Genotype-Tissue Expression Program (GTEx) databases using Gene Expression Profiling Interactive Analysis (GEPIA). GSE86374 and GSE58135 datasets from the Gene Expression Omnibus (GEO) database were also used for this purpose. We performed survival analysis based on CENPF expression in breast cancer patients, especially patients treated with neoadjuvant chemotherapy, using Kaplan‒Meier Plotter with the best-performing threshold as a cut-off. We downloaded data on TNBC patients from the GSE25066 dataset and used Sangerbox to analyse the relationship between the expression level of CENPF and patient prognosis with the best-performing threshold as a cut-off.

### Patient samples and IHC

TNBC tissue blocks were collected from 32 patients operated on at Qilu Hospital and The Second Hospital of Shandong University between 2013 and 2020. All 32 patients received at least four cycles of neoadjuvant chemotherapy before surgical resection. Tumour grade and MP grade were determined as previously described^16^. According to the MP grading system, grade 3 to grade 5 tumours were classified into the chemotherapy-sensitive group, and grade 1 and grade 2 tumours were regarded as chemotherapy-resistant. IHC and evaluation of immunohistochemical (IHC) staining were performed as previously described^16^.

### Cell lines and cell culture

We purchased the human breast cancer cell line MDA-MB-231 from the American Type Culture Collection (ATCC; Manassas, VA, USA). Cell authentication was verified by short tandem repeat profiling. The MDA-MB-231 cell line with adriamycin resistance (MDA-MB-231/ADR) was induced by long-term adriamycin treatment at the laboratory in the early stage and was tested for adriamycin resistance^8^. We cultured these cell lines in Leibovitz’s L15 medium (MDA-MB-231 and MDA-MB-231/ADR) supplemented with 10% foetal bovine serum at 37 °C with 5% CO2. All cell lines were determined to be free from mycoplasma contamination by mycoplasma PCR testing and no more than 20 cell passages were allowed for any experiment.

### Antibodies and reagents

Anti-phospho-Chk1-Ser317 antibody (1:1000, 12302), anti-phospho-Chk1-Ser345 antibody (1:10002348), and anti-Rb antibody (1:500, 9309) were purchased from Cell Signaling Technology (CST, Danvers, Massachusetts, US). Anti-CENPF antibody (1:1000, DF2310), anti-Chk1 antibody (1:1000, AF6007), anti-E2F1 antibody (1:500, DF6797) and anti-GAPDH antibody (1:3000, AF7021) were purchased from Affinity Biotech (Cincinnati, Ohio, US). Anti-CENPF antibody (1:1000, ab5) was purchased from Abcam (Cambridge, UK).

### Cell transfection and siRNAs

Both si-CENPF and si-Control were synthesized by GenePharma (Shanghai, China) according to the sequence verified by Sigma Aldrich. We used Lipofectamine 3000 (Invitrogen, Carlsbad, CA, USA) and transfected MDA-MB-231 and MDA-MB-231/ADR cells with siRNA (50 nM) according to the manufacturer's protocol. We confirmed the knockdown efficiency of CENPF by RT‒qPCR and western blotting 48 h after cell transfection. All siRNA sequences are shown in Supplementary Table S1.

**Figure 1:**
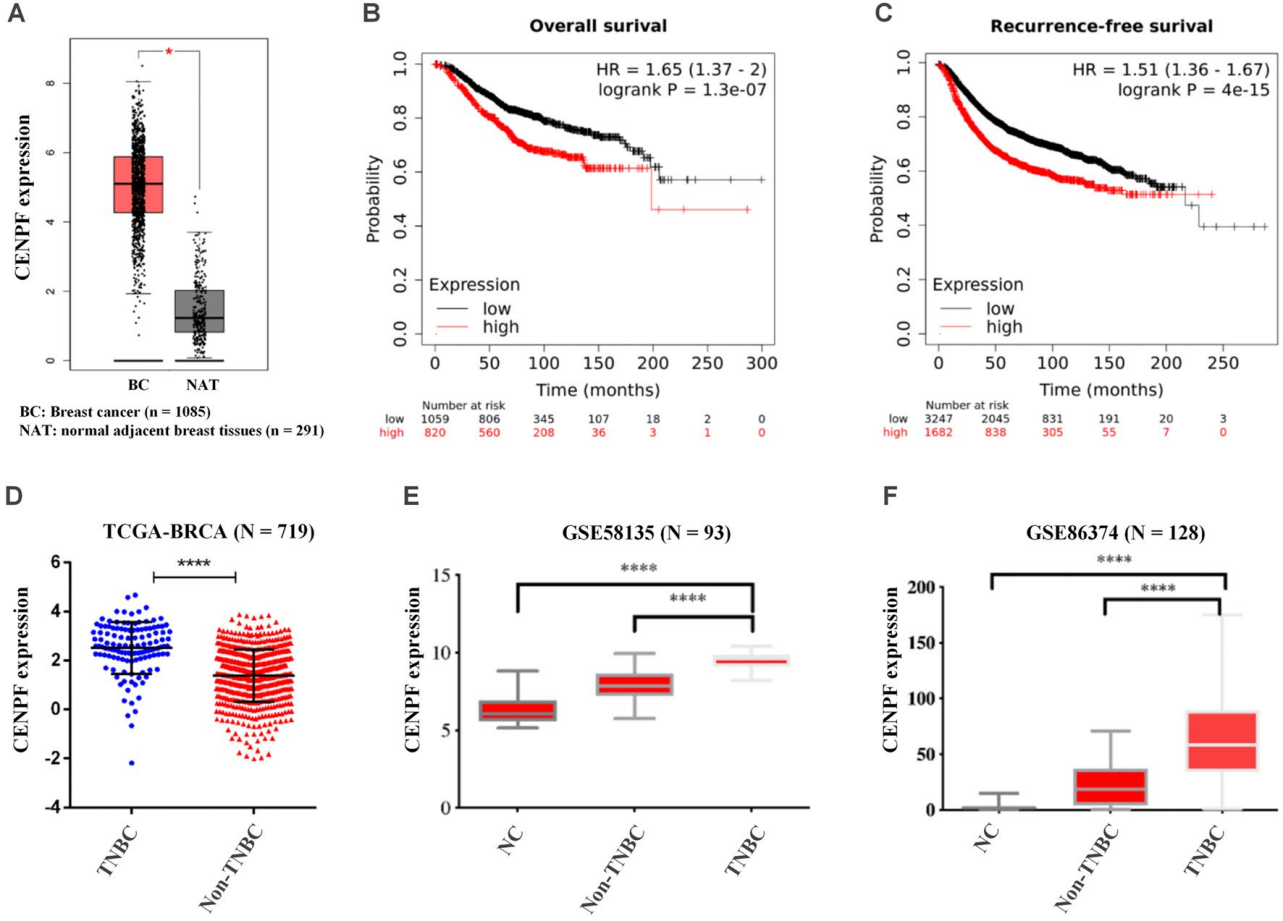
CENPF is highly expressed in triple-negative breast cancer. (**A**) CENPF expression in breast cancer tissues (n = 1,085) and adjacent peritumoral tissues (n = 291) from the TCGA and GTEx databases using GEPIA (http://gepia.cancer-pku.cn/index.html). (**B**,**C**) Overall survival (**B**, n = 1 879, log-rank P = 1.3e−07) and recurrence-free survival (**C**, n = 4929, log-rank P < 1e−15) of breast cancer patients. Data were obtained from Kaplan‒Meier Plotter (http://kmplot.com/analysis/). (**D**–**F**) Based on data from the TCGA (**D**), GSE58135 (**E**) and GSE86374 (**F**), CENPF expression in TNBC and non-TNBC samples. Data shown represent the means (± standard deviation [SD]); *P < 0.05, ****P < 0.0001; log-rank test (**B**,**C**), Student’s t test (**D**) or one-way ANOVA (**E**,**F**).

### Quantitative real-time PCR (qRT‒PCR)

Total RNA and RT‒qPCR were performed as previously described^17^. All primer sequences are presented in Supplementary Table S2.

### Western blotting

Cells were lysed on ice for 20 min in RIPA lysis buffer (Beyotime, Beijing, China) supplemented with 1% phenylmethanesulfonyl fluoride (Beyotime, Beijing, China) and 1% ProtLytic Phosphatase Inhibitor Cocktail (New Cell and Molecular Biotech, Suzhou, China). The solution was then centrifuged at 12,000*g* for 10 min, and the supernatants were collected. Next, the total protein concentration was measured using a BCA kit (Invitrogen, Carlsbad, CA, USA). After denaturation at 100 °C for 8 min, 20 μg protein samples were separated on 7.5% SDS/PAGE gels and then transferred to PVDF membranes (Merck Millipore, Billerica, MA, USA).

### Drug sensitivity assay

A total of 5000 cells were seeded in 96-well plates. After 24 h, the medium was changed to medium containing adriamycin (MB1087, Dalian Meilun Bio, Dalian, China), and the culture was continued for 72 h at drug concentrations of 0, 0.1, 0.5, 1, 2, 5, 10 and 20 μM. Cell viability was assessed using Cell Counting Kit-8 (CCK-8; K1018A, PExBIO, Houston, TX, USA) following the manufacturer's instructions. After 2 h of incubation at 37 °C, the absorbance was read at 450 nm.

### Cell proliferation assay

Cells (3,000) were seeded in 96-well plates. Cell viability was assessed using Cell Counting Kit-8 (CCK-8; K1018A, PExBIO, Houston, TX, USA) following the manufacturer's instructions at 24, 48, 72, and 96 h. After 2 h incubation at 37 °C, absorbance was read at 450 nm. Furthermore, EdU cell proliferation assays were further confirmed as previously described^18^.

### Cell apoptosis assay

We collected MDA-MB-231 and MDA-MB-231/ADR cells transfected with si-CENPF-1, si-CENPF-2 or si-control after 48 h of exposure to ADR at a concentration of 1 or 5 μM, which was consistent with the process for the ADR-free group. Annexin V-fluorescein isothiocyanate (FITC) and propidium iodide (PI) staining were performed using the Annexin V-FITC/PI Apoptosis Assay Kit (BestBio, Shanghai, China) according to the manufacturer's instructions. Apoptosis was then detected immediately by flow cytometry (Cytoflex S, Beckman Coulter, California, US).

### Cell cycle analysis

We collected MDA-MB-231 cells transfected with si-CENPF-1, si-CENPF-2 or si-control after 48 h of exposure to ADR at a concentration of 1 μM, which was consistent with the process for the ADR-free group. Staining was performed using the Cell Cycle Detection Kit (BestBio, Shanghai, China) according to the manufacturer's instructions, and flow cytometry (Cytoflex S, Beckman Coulter, CA, USA) was performed to determine the effects of CENPF inhibition on cell cycle distribution with or without 48 h of treatment with 1 μM adriamycin in MDA-MB-231 cells. Histograms were used to statistically analyse the cell cycle distribution.

### Immunofluorescence

Cells were cultured on glass slides and washed twice with PBS before fixation. Cells were fixed with 4% paraformaldehyde for 15 min and permeabilized with 0.1% Triton X-100 in PBS for 10 min at room temperature. To reduce nonspecific binding of antibodies, cells were incubated in PBS containing 1% BSA for 1 h at room temperature. Primary antibodies (anti-CENPF and anti-Rb) were diluted in PBS containing 1% BSA, dropped on cells and incubated overnight at 4 °C. Next, the samples were incubated with secondary antibodies DyLight488 and DyLight594 (Beyotime, Beijing, China), which were diluted in PBS containing 1% BSA for 1 h at room temperature. Then, antifade mounting medium with DAPI (Beyotime, Beijing, China) was added dropwise, and images were observed and collected under a fluorescence microscope.

### Coimmunoprecipitation

Cell lysis and protein concentration determination were performed as described above. For immunoprecipitation, equal amounts of lysate were incubated with protein A/G magnetic beads (Beyotime, Beijing, China) and anti-CENPF antibody overnight at 4 °C. Thereafter, the beads were washed 3 times with cell lysis buffer, and immunoprecipitated proteins were analysed by western blotting.

### Statistical analysis

Statistical analysis was performed using GraphPad Prism 5 (GraphPad Software, Inc., San Diego, California, US, https://www.graphpad.com/) and SPSS 16.0 (SPSS Inc., IL, USA, https://www.ibm.com/cn-zh/spss), and a t test was used to determine the significance fo differences between two independent samples. One-way ANOVA was used to analyse the significance of differences between three or more independent samples. Analysis of the relationship between CENPF expression and neoadjuvant chemotherapy grade and clinicopathological parameters was performed using the chi-square test and Fisher's exact test. We used the Kaplan‒Meier method and log-rank t test to determine the significance of differences in survival curves. Correlation analysis between coexpressed genes in the TCGA dataset in breast cancer was performed using Pearson's correlation coefficient; immunofluorescence colocalization of CENPF and Rb proteins analysed using Pearson's correlation coefficient, R values > 0.3 or < -0.3 were considered to indicate statistical significance. Each experiment was repeated at least 3 times, and P < 0.05 was considered to indicate statistical significance.

### Approval of the research protocol by an Institutional Reviewer Board

Ethics Committee of School of Basic Medical Sciences, Shandong University. All methods were performed in accordance with the relevant guidelines and regulations.

### Informed consent

All informed consent was obtained from the subject(s) and/or guardian(s).

## Results

### CENPF is highly expressed in triple-negative breast cancer

The published data from the TCGA showed that CENPF expression in breast cancer tissues was significantly higher than that in adjacent peritumoral tissues (Fig. 1A, n = 1 376, P < 0.05). Survival prognostic analysis using the Kaplan‒Meier Plotter tool showed that low levels of CENPF expression predicted better overall survival (Fig. 1B, n = 1 879, log-rank P = 1.3e − 07) and recurrence-free survival (Fig. 1C, n = 4929, log-rank P < 1e − 15) in breast cancer. Moreover, based on published data from the TCGA and GEO (GSE58135 and GSE86374), we compared CENPF expression between non-TNBC and TNBC. This result suggested that high CENPF expression was more pronounced in TNBC (Fig. 1D–F).

### CENPF increases the ADR chemosensitivity of TNBC

**Figure 2:**
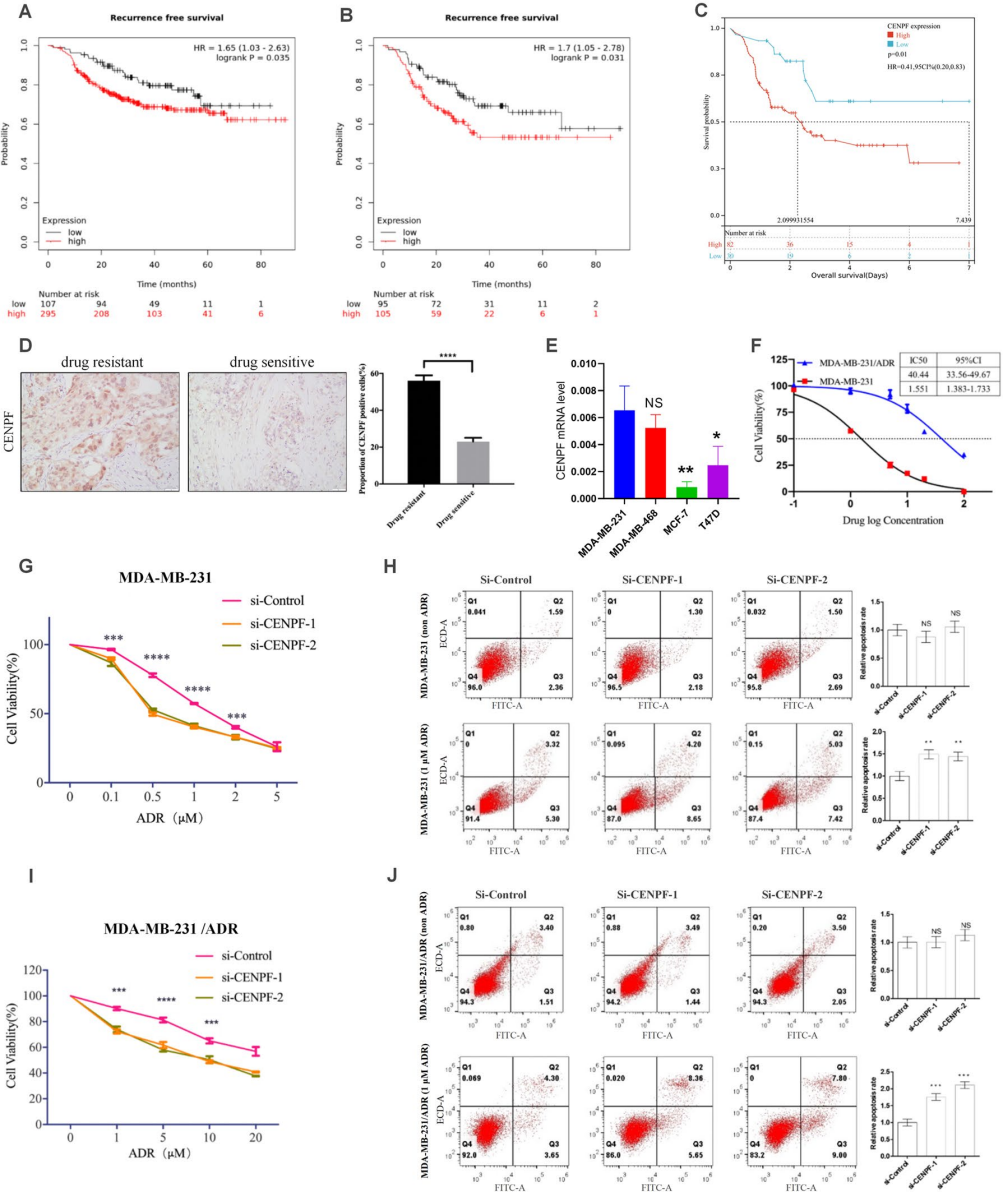
CENPF increases the ADR chemosensitivity of TNBC. (**A,B**) Recurrence-free survival in breast cancer patients receiving NACT (**A**, n = 403, log-rank P = 0.035) or TNBC treatment with NACT (**B**, n = 200, log-rank P = 0.031), using Kaplan‒Meier Plotter. (**C**) Overall survival in TNBC patients receiving NACT (**C**, n = 112, P = 0.02). Data were obtained from GSE25066. (**D**) Quantification of IHC staining for CENPF chemo-resistant and chemotherapy-sensitive TNBC. (**E**) CENPF mRNA quantification in MDA-MB-231, MDA-MB-468, MCF-7 and T47D cells performed by RT‒qPCR. F IC50 of adriamycin in MDA-MB-231 and MDA-MB-231/ADR cells. (**G**–**J**) We used a drug sensitivity assay to assess the effects of CENPF knockdown on ADR chemosensitivity in MDA-MB-231 (**G**) and MDA-MB-231/ADR (**I**) cells. We performed an Annexin V-FITC/PI apoptosis assay to investigate the role of CENPF in cell apoptosis with or without adriamycin in MDA-MB-231 (H) and MDA-MB-231/ADR (**J**) cells. Data shown represent the means (± SD) of three independent experiments; **P < 0.01, ***P < 0.001, ****P < 0.0001; NS, not significant; log-rank test (**A**–**C**), nonlinear regression (**F**) or one-way ANOVA (**G**–**J**).

Kaplan‒Meier plotter data showed that high CENPF expression portended worse recurrence-free survival in breast cancer patients receiving neoadjuvant chemotherapy (NACT) (Fig. 2A, n = 403, log-rank P = 0.035). Chemotherapy is the main strategy for the treatment of TNBC, but its frequent resistance to chemotherapy is a significant clinical challenge. Survival analysis suggested that TNBC with high CENPF expression had a worse prognosis after treatment with neoadjuvant chemotherapy (Fig. 2B, n = 200, log-rank P = 0.031). This point was also supported by the published data from GSE25066 (Fig. 2C, n = 112, P = 0.02). To further confirm the relationship between CENPF and chemotherapy resistance, we collected puncture specimens from 32 cases of TNBC before neoadjuvant chemotherapy in recent years. They were further stratified by assessment of chemotherapy response according to the Miller and Payne (MP) grading system. According to reported studies^19^, patients with tumours rated as Miller-Payne regression grades 1 and 2 were assigned to the chemoresistant group, and those with tumour Miller-Payne regression grades 3–5 were considered the chemotherapy-sensitive group. The results of immunohistochemical staining indicated that the chemoresistant group displayed significantly higher CENPF expression than the chemotherapy-sensitive group (Fig. 2D). As shown in Table 1, the results of the analysis grouped according to CENPF expression also showed that high CENPF expression was associated with chemotherapy resistance.

**Table 1:** Clinicopathological characteristics of 32 TNBC.

Variable	Cases	CENPF expression		P value
		Low	High	
Age				
< 50 years	15	6	9	0.688
≥ 50 years	17	8	9	
Tumor diameter				
≤ 3 cm	15	9	6	0.082
> 3 cm	17	5	12	
Lymph node metastasis				
Negative	12	5	8	0.710
Positive	20	9	11	
Drug resistance				
Chemoresistance	22	7	15	**0.0435**
Chemosensitive	10	7	3	
P53				
Negative	10	6	4	0.334
Positive	16	5	11	
Missing	6	3	3	

*The intensity of nuclear staining was scored and graded on a 0–3 scale: 0 (negative); 1 + (weak positive), 2 + (moderate positive) and 3 + (strong positive). This initial score was further classified as low expression (0–1) or high expression (2–3). P value was calculated by statistical analysis of Pearson's Chi-square test. Significant values are in bold.

Consistent with the assay results from tissues, CENPF expression was significantly increased in TNBC cell lines (MDA-MB-231 and MDA-MB-468) compared to non-TNBC cell lines (MCF-7 and T47D) (Fig. 2E). MDA-MB-231 and adriamycin-resistant strains (MDA-MB-231/ADR) were used for subsequent research on the mechanism by which CENPF is involved in chemotherapy resistance in TNBC. Establishment of MDA-MB-231/ADR cells was accomplished in our previous study^8^. The adriamycin resistance assays of the two cell lines are shown in Fig. 2F. Two specific interference sequences were designed to inhibit CENPF. The interference efficiency of si-CENPF-1 and si-CENPF-2 was analysed via western blotting and RT-qPCR after 48 h of transfection (Supplementary Fig. S1A–D). The CENPF mRNA expression in MDA-MB-231 and MDA-MB-231/ADR was analysed via RT-qPCR (Supplementary Fig. S1E). CENPF knockdown potentiated ADR-induced cytotoxicity in MDA-MB-231 cells (Fig. 2G). Using flow cytometry, we found that silencing CENPF increased the ADR-induced apoptosis rates of MDA-MB-231 cells (Fig. 2H). As shown in Fig. 2I,J, these findings were consistent with those in MDA-MB-231/ADR cells.

### CENPF regulates ADR-induced G2/M phase arrest through Chk1

**Figure 3:**
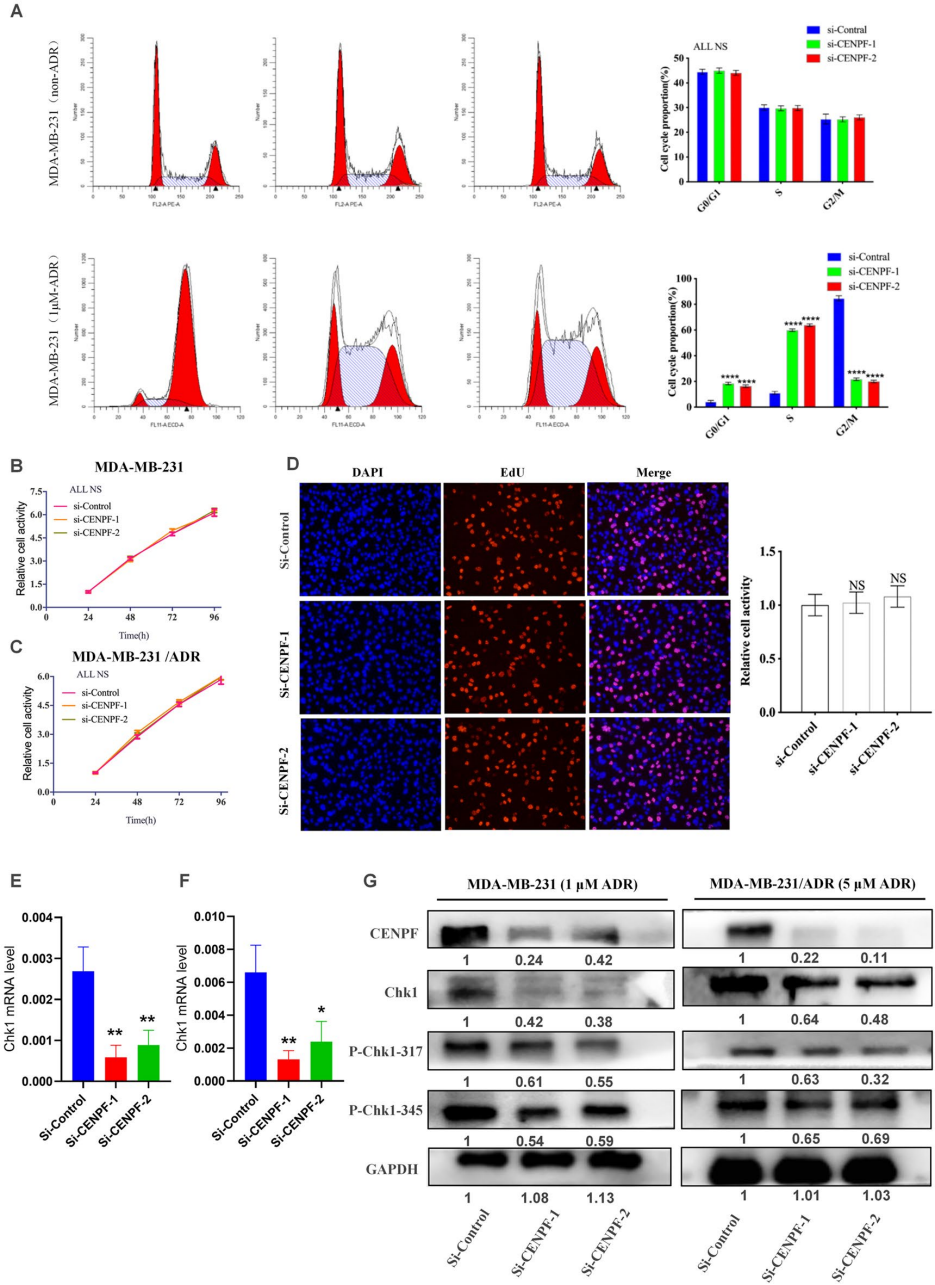
CENPF regulates ADR-induced G2/M phase arrest through Chk1. (**A**) Flow cytometry was performed to determine the effects of CENPF inhibition on cell cycle distribution with or without adriamycin in MDA-MB-231 cells. (**B**,**C**) The effect of CENPF inhibition on cell proliferation in MDA-MB-231 (**B**) or MDA-MB-231/ADR (**C**) cells. (**D**) Cell proliferation was determined by EdU assay. (**E**,**F**) Using RT‒qPCR, the mRNA level of CHK1 was detected in MDA-MB-231 (**E**) or MDA-MB-231/ADR (**F**) cells with CENPF knockdown. (**G**) Western blot analysis of the effect of CENPF knockdown on Chk1 expression and phosphorylation in MDA-MB-231 and MDA-MB-231/ADR cells exposed to adriamycin. Data shown represent the means (± SD) of three independent experiments; **P < 0.01, ****P < 0.0001; *NS* not significant; one-way ANOVA (**A**–**F**).

CENPF is strictly expressed in a cell cycle-dependent manner, and it starts to accumulate in the S phase and peaks in the G2/M phase. To reveal the mechanism by which CENPF promotes chemotherapy resistance, we used flow cytometry to observe the effects of CENPF inhibition on cell cycle distribution. Adriamycin induced significant G2/M arrest, which was reversed by CENPF inhibition. As shown in Fig. 3A, CENPF knockdown significantly decreased the number of cells in G2/M phase and increased the number of cells in G0/G1 and S phases under adriamycin treatment. Interestingly, our findings suggested that CENPF lost the control over cell cycle in TNBC without exogenous stress (Fig. 3A). This was also confirmed by cell proliferation assays using CCK-8 (Fig. 3B,C) and EdU (Fig. 3D). The above results suggested that CENPF is required to cope with adriamycin-induced DNA damage stress in TNBC.

Our previous study showed that under DNA damage stress, Chk1 is activated in a phosphorylated form to facilitate DNA repair by inducing G2/M phase arrest in TNBC^8^. In the DNA damage response (DDR), Chk1-induced cell cycle arrest provides sufficient time for DNA repair to prevent DNA damage from causing apoptosis^20^. The results of RT‒qPCR (Fig. 3E,F) and western blotting (Fig. 3G) showed that CENPF knockdown inhibited Chk1 expression and thus impaired Chk1 activation in MDA-MB-231 and MDA-MB-231/ADR cells exposed to adriamycin. Finally, CENPF regulates ADR-induced G2/M phase arrest through Chk1 to regulate the ADR chemosensitivity of TNBC.

### CENPF alters the transcriptional activity and expression of E2F1

**Figure 4:**
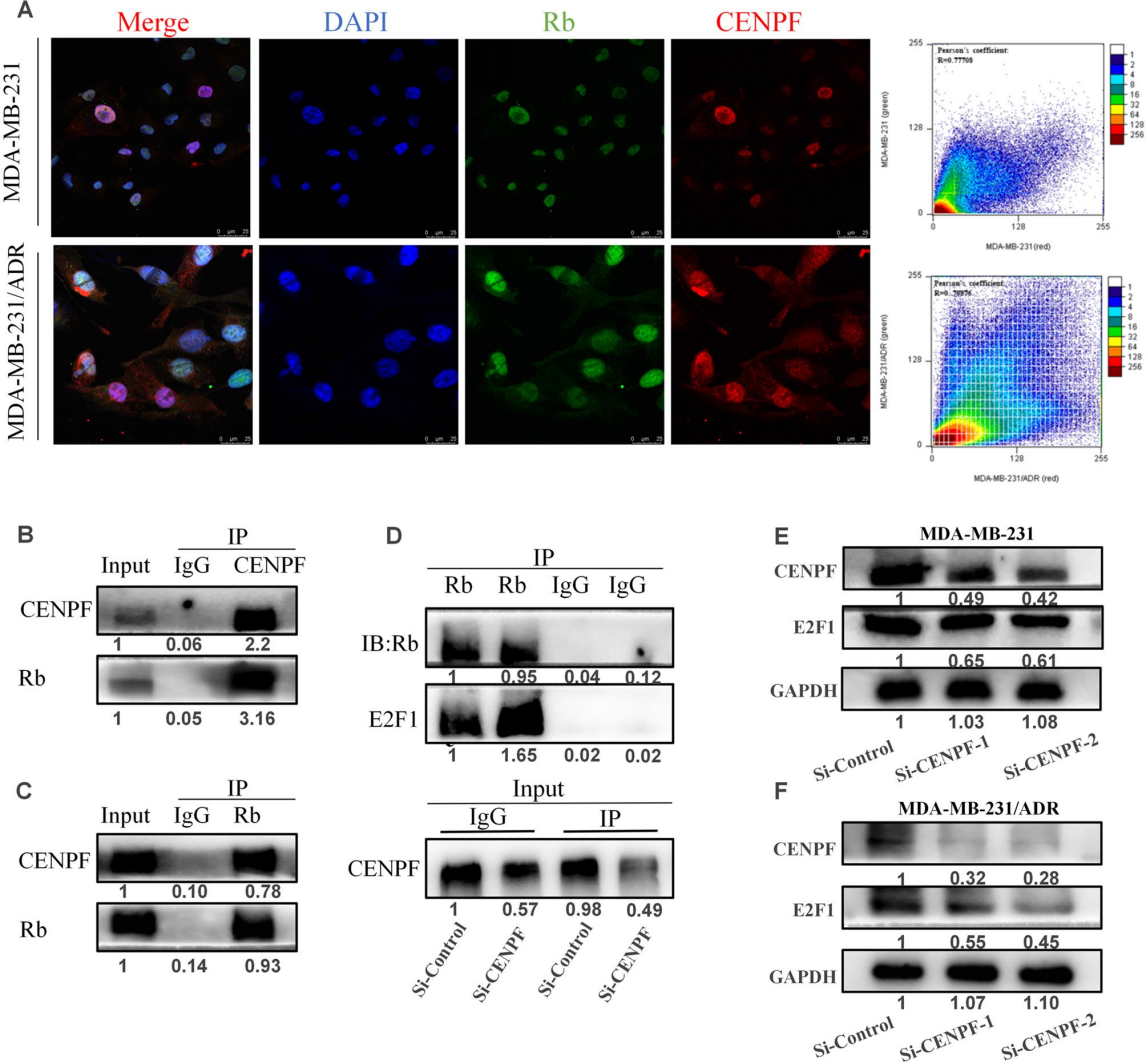
The Rb-E2F1 axis mediates the regulation of Chk1 expression by CENPF. (**A**) Colocalization of CENPF and Rb in MDA-MB-231 and MDA-MB-231/ADR cells. (**B**,**C**) Western blotting detected the binding protein of immunoprecipitated endogenous CENPF (B) or Rb (Rb). (**D**) Co-IP and western blotting demonstrated that CENPF and E2F1 compete for Rb binding. (**E**,**F**) Western blot analysis of the effect of CENPF knockdown on E2F1 expression.

According to reports, CENPF orthologues LEK1 and CMF1 function in muscle differentiation through interaction with Rb. They bind Rb in vitro through a region homologous to that of E2F1^21^. Bargiela-Iparraguirre et al. found that Chk1 expression is controlled by Rb/E2F1 at the transcriptional level^22^. Considering the crucial role of E2F1 in the DNA damage response and DNA repair^23,24^, we assumed that CENPF is likely to target the Rb-E2F1 axis to regulate TNBC chemoresistance. Confocal images showed colocalization of CENPF with Rb in MDA-MB-231 and MDA-MB-231/ADR cells (Fig. 4A). To further validate the interaction of CENPF and Rb, we performed a coimmunoprecipitation assay, which showed that immunoprecipitated endogenous CENPF bound to endogenous Rb (Fig. 4B). Immunoprecipitation of endogenous Rb led to the coprecipitation of CENPF (Fig. 4C). Moreover, as shown in Fig. 4D, CENPF knockdown increased the binding of Rb to E2F1, thereby inhibiting E2F1 release. In addition, CENPF knockdown significantly decreased E2F1 expression in MDA-MB-231 (Fig. 4E) and MDA-MB-231/ADR cells (Fig. 4F).

## Discussion

Cell cycle arrest and DNA repair are essential components of the DDR, which is required to maintain the genomic integrity of cancer cells, especially when exposed to exogenous DNA-damaging agents^25,26^. These protective DDR processes become direct drivers of tumour chemoresistance. TNBC patients often develop primary or acquired chemoresistance, leading to chemotherapy failure, recurrence and even much shorter survival time. In this study, we found that the Rb-E2F1 axis mediates the regulation of Chk1 expression by CENPF and that CENPF promotes adriamycin chemoresistance by regulating Chk1-mediated G2/M phase arrest in TNBC. CENPF is a new potential intervention target to overcome chemoresistance in TNBC.

At present, the mechanism of CENPF in the chemotherapy resistance of TNBC remains unclear. Our study shows that CENPF, upstream of Chk1, targets and regulates ADR-induced G2/M phase arrest. Chk1 is one of the key proteins of the G2/M phase checkpoint. It is involved in the DNA damage response caused by endogenous and exogenous factors and is also critical for genome stability and cell survival^27^. In DNA damage stress, transient cell cycle arrest is a favourable event for cell survival because it provides sufficient time and space for DNA repair^28^. Once DNA repair is disrupted, the main process will switch from cell cycle arrest to apoptosis^29^. Our previous studies have shown that Chk1 inhibition enhances the adriamycin chemosensitivity of TNBC by eliminating cell cycle arrest and inducing cell apoptosis^8^. TNBC often exhibits dysregulation of G1/S cell cycle checkpoints, so it is more dependent on G2/M phase checkpoints for DNA repair and maintenance of chromosomal integrity. Therefore, our results suggest that CENPF knockdown ultimately promotes adriamycin-induced cell apoptosis as G2/M phase arrest is counteracted.

Then, we found that CENPF binds to Rb and competes with the E2F1 binding site. Rb binds to E2F1 and inhibits the transcriptional activity of E2F1 to regulate the cell cycle, which has been extensively studied^30^. As our results showed, CENPF knockdown increased the binding of Rb to E2F1, thereby inhibiting E2F1 release. Transcription factor E2F1, a member of the E2F family, plays an important role in the regulation of cell cycle progression, DNA repair, apoptosis and drug resistance^24,31^. Verlinden et al. confirmed that the E2F1 transcription factor aggregates at the Chk1 promoter and upregulates Chk1 expression in TNBC^32^. Bargiela-Iparraguirre et al. demonstrated that Chk1 overexpression specifically increases resistance to chemo/radiotherapy in gastric cancer and that Chk1 expression is controlled by Rb/E2F1 at the transcriptional level^22^. In summary, the Rb-E2F1 axis mediates the regulation of Chk1 expression by CENPF, which contributes to chemotherapy resistance in TNBC.

In addition, the oncogenic role of E2F1 in mediating cancer chemoresistance has been confirmed by multiple previous studies^33,34^. The transcription factor E2F1 has major roles in cell cycle control, checkpoint response and DNA replication and repair, which leads to DNA damage tolerance in tumour cells^23,24^. Our findings on the regulation of CENPF on the transcriptional activity and expression of E2F1 revealed a new mechanism by which CENPF is involved in TNBC chemoresistance. Notably, although CENPF knockdown increased the binding of Rb to E2F1, it had no effect on cell cycle progression and cell proliferation under normal growth conditions. Here, we focused on Igor Shats et al.’s studies, which demonstrated that E2F1-mediated cell fate depends on its expression level, and low levels of E2F1 facilitate proliferation, while high levels facilitate apoptosis^35^. And the deregulation of the cell cycle is a common state for TNBC^36^. In the absence of exogenous DNA damage, these tumour cells tend to escape from cell cycle checkpoint for unlimited proliferation.

Due to time and technical limitations, there are several shortcomings to the present study. To ensure that paraffin sections meet the requirements of IHC detection, most of the specimens we collected are from recent cases, so the follow-up results cannot be presented. Considering this issue, we utilized the TCGA and GEO databases for prognostic analysis, as shown in Figs. 1 and 2. In addition, the molecular weight of CENPF, as high as 360 kDa, is too high to perform in vitro CENPF overexpression assays.

Previous studies on CENPF have mainly focused on kinetochore function, tumour proliferation and metastasis^37–39^. Here, we demonstrate a novel mechanism by which CENPF regulates TNBC chemoresistance through the Rb-E2F1-Chk1 axis. Our findings are the first to indicate CENPF as a potential target for intervention to overcome chemotherapy resistance in TNBC. We believe that further research on the mechanism of CENPF will provide new ideas for addressing TNBC chemoresistance.

## Supplementary Information

Supplementary Information.

## Data Availability

The datasets analysed during the current study are available in the [GEO] repository, [https://www.ncbi.nlm.nih.gov/geo/] and the [TCGA] repository, [https://www.cancer.gov/about-nci/organization/ccg/research/structural-genomics/tcga].
