# Supplementary material for: CENPF knockdown inhibits adriamycin chemoresistance in triple-negative breast cancer via the Rb-E2F1 axis
Source: Sci Rep. 2023 Jan 31;13:1803. doi: 10.1038/s41598-023-28355-z (PMC9889717; doi:10.1038/s41598-023-28355-z)

**Supplementary Fig. 1**. The Western blotting result of knockdown efficiency about si-CENPF-1 and si-CENPF2 in MDA-MB-231 (A) and MDA-MB-231/ADR (B) cells. The RT-qPCR result of knockdown efficiency about si-CENPF-1 and si-CENPF2 in MDA-MB-231 (C) and MDA-MB-231/ADR (D) cells. The RT-qPCR result of CENPF mRNA expression in MDA-MB-231 and MDA-MB-231/ADR (E).

**Supplementary Table 1 Sequences of siRNAs.**

| Sequences of siRNA | Sequence (5’→3’) |
| --- | --- |
| si-control | UUCUCCGAACGUGUCACGUTT |
| CENPF-siRNA-1 | CCCAAGAGAAUGGGACUCUUA |
| CENPF-siRNA-2 | GCGAGUCAGAUCAAGGAGAAU |

**Supplementary Table 2 List of primers used for quantitative real time-PCR.**

| Gene Symbol | Sequence (5’→3’) |
| --- | --- |
| GAPDH forward | TGCACCACCAACTGCTTAGC |
| GAPDH reverse | GGCATGGACTGTGGTCATGAG |
| CHK1 forward | AAGACTGGGACTTGGTGCAA |
| CHK1 reverse | CAGTCTACGGCACGCTTCAT |
| GENPF forward | TGCACAAAGACCAGGTGGAAA |
| GENPF reverse | GTCCAAAAGCAAAGCCTGGTG |

**original data**

We provide images showing full-length membranes, with membrane edges visible. Some images of the blots were cut prior to hybridization with antibodies, therefore the images in this file is the full-length membranes. We have denoted the regions of the original blots used in the main figures using red boxes

Fig. 3G MDA-MB-231 (1μM ADR) CENPF


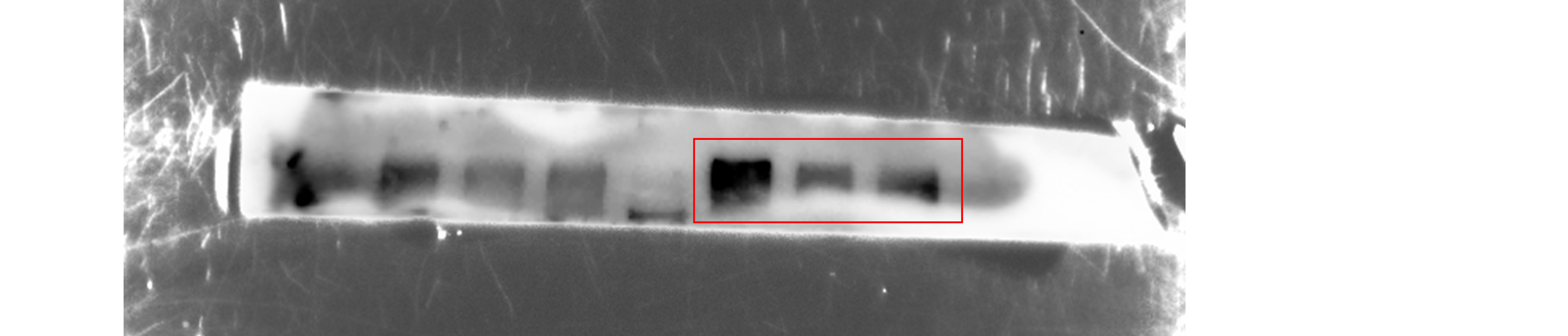


Fig. 3G MDA-MB-231 (1μM ADR) Chk1


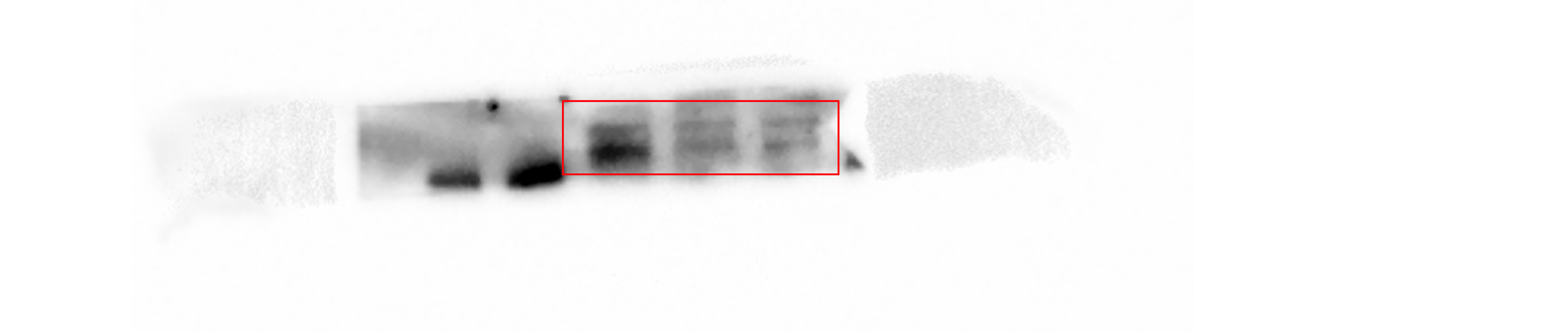


Fig. 3G MDA-MB-231 (1μM ADR) P-Chk1317


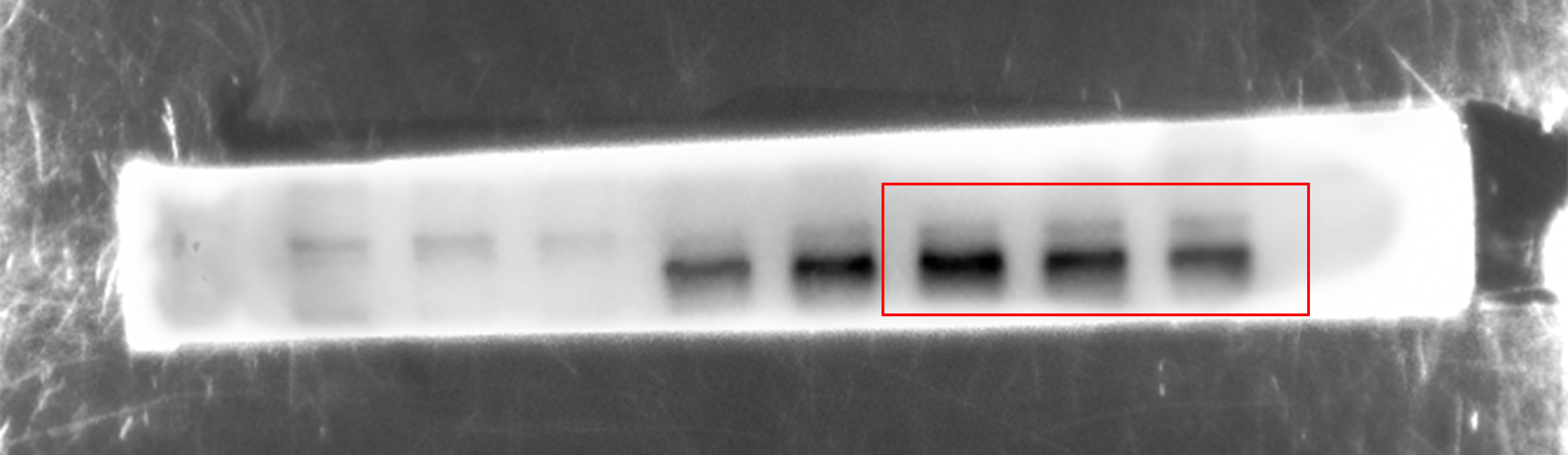


Fig. 3G MDA-MB-231 (1μM ADR) P-Chk1345


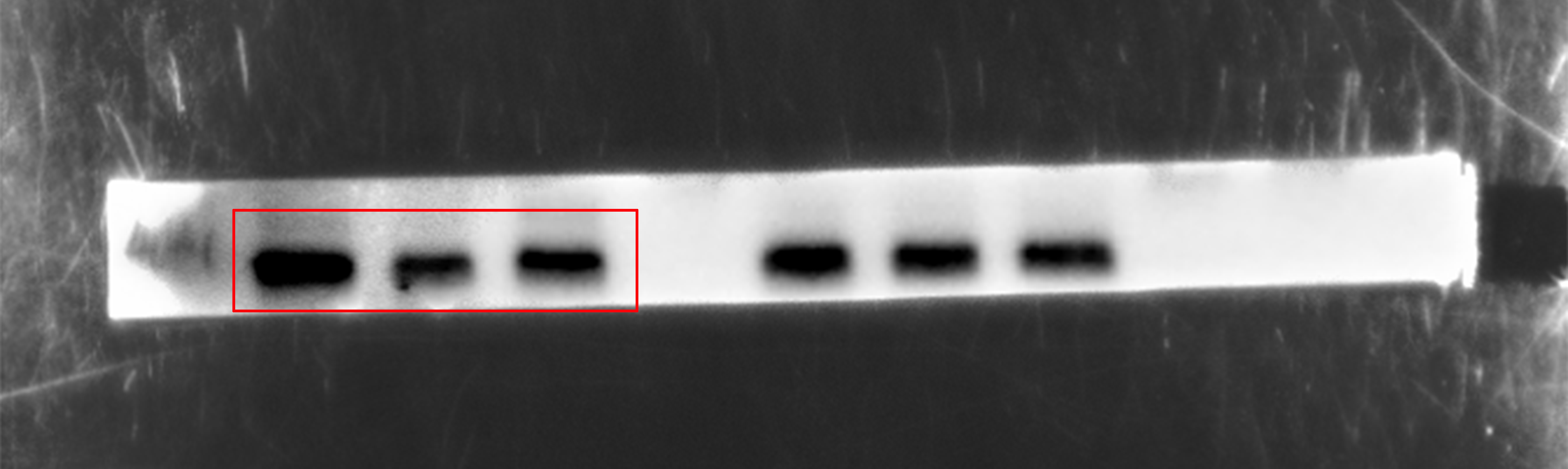


Fig. 3G MDA-MB-231 (1μM ADR) GAPDH


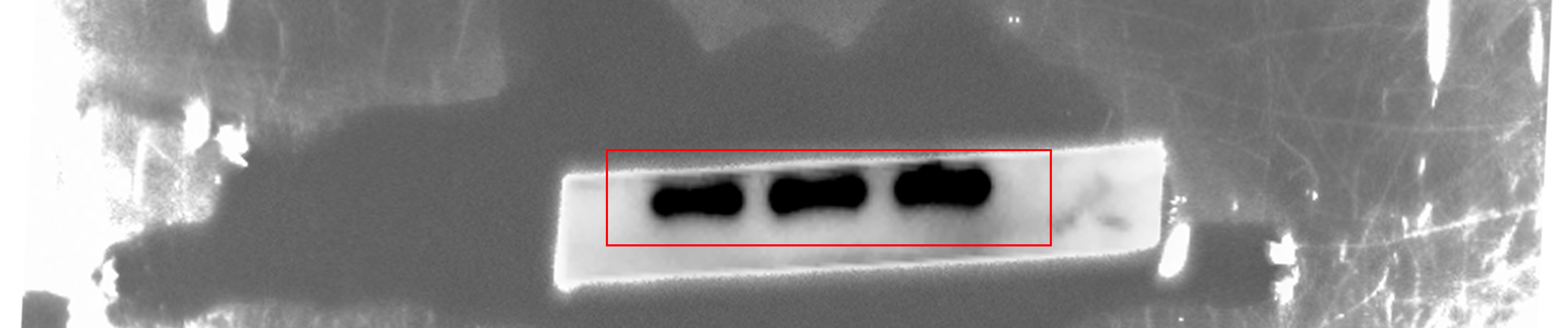


Fig. 3G MDA-MB-231 (5μM ADR) CENPF


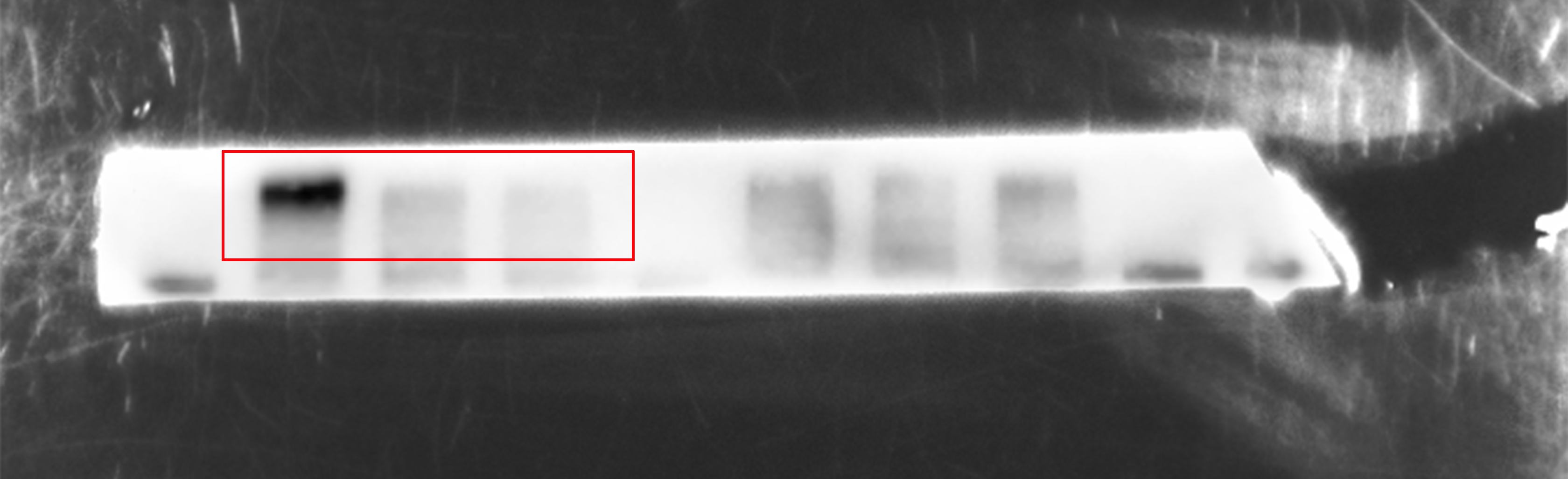


Fig. 3G MDA-MB-231 (5μM ADR) Chk1


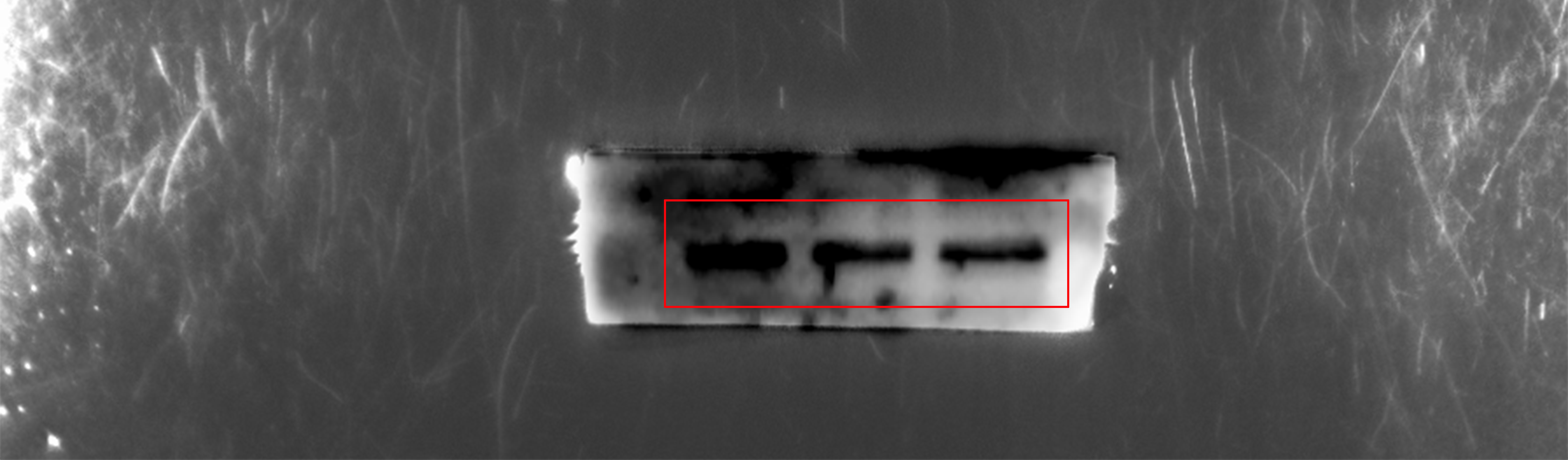


Fig. 3G MDA-MB-231 (5μM ADR) P-Chk1317


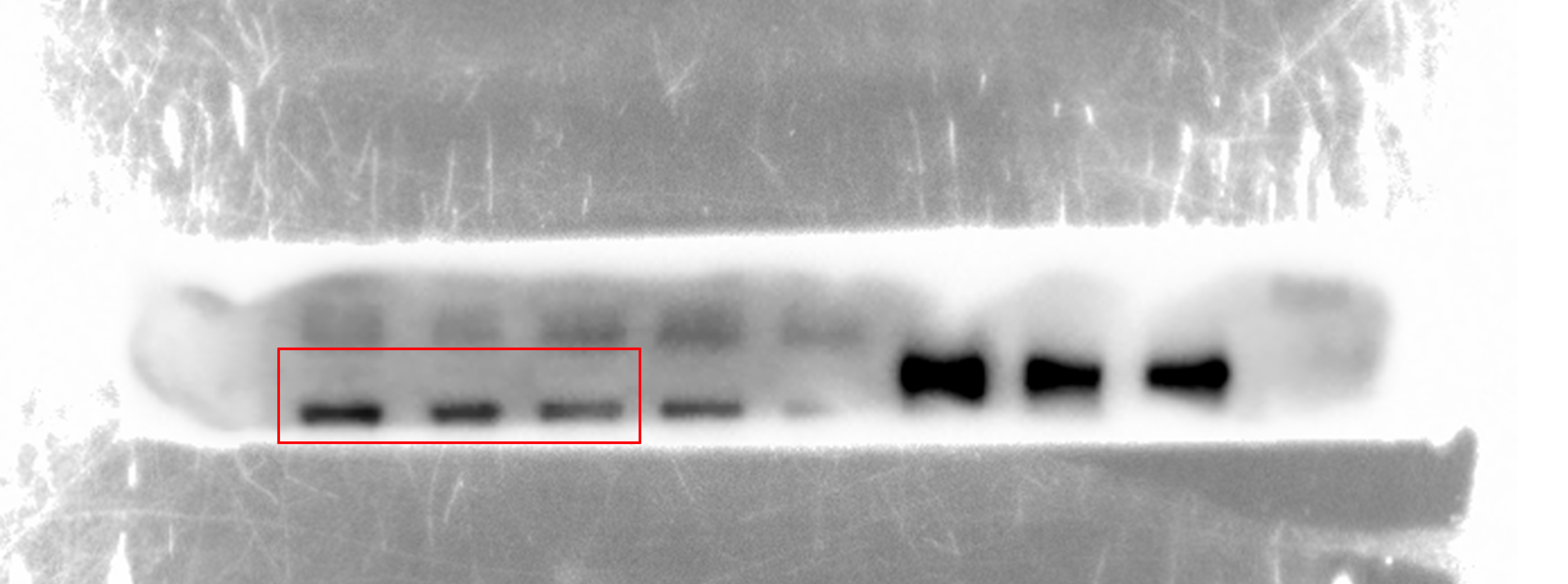


Fig. 3G MDA-MB-231 (5μM ADR) P-Chk1345


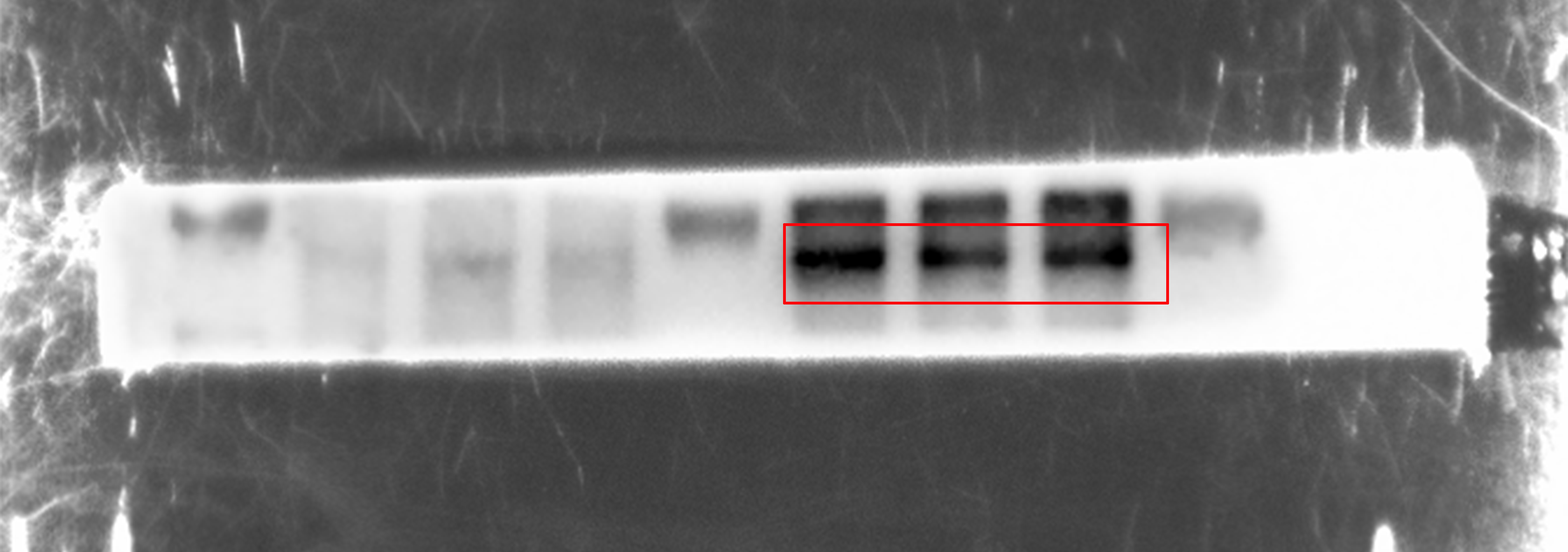


Fig. 3G MDA-MB-231 (5μM ADR) GAPDH


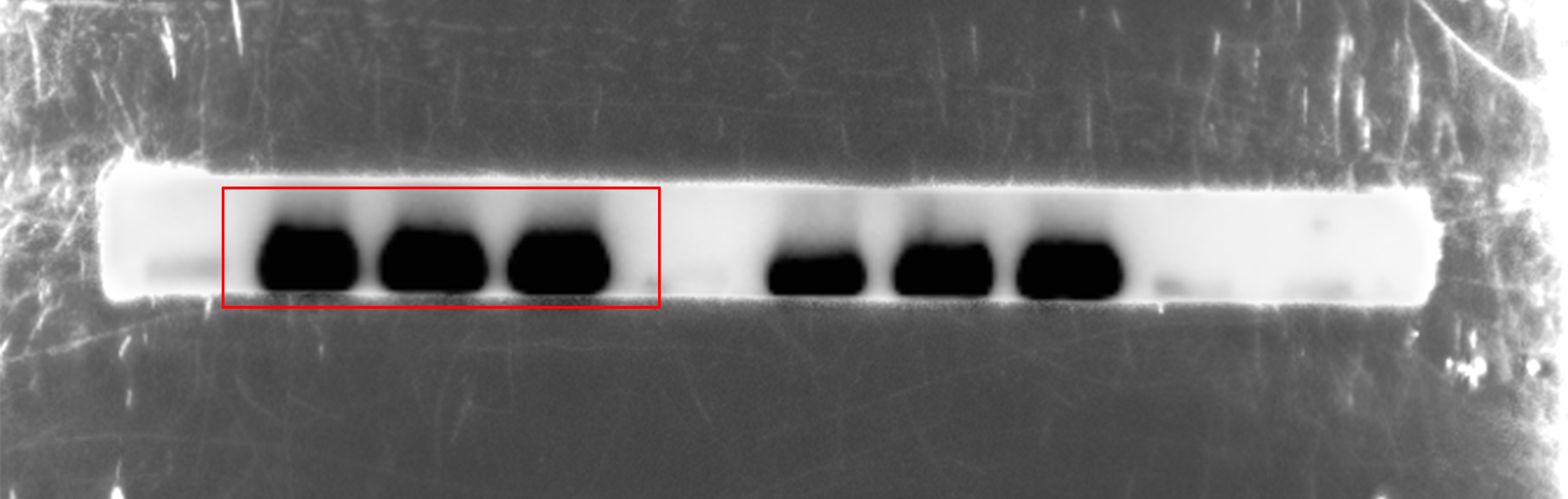


Fig. 4B CENPF


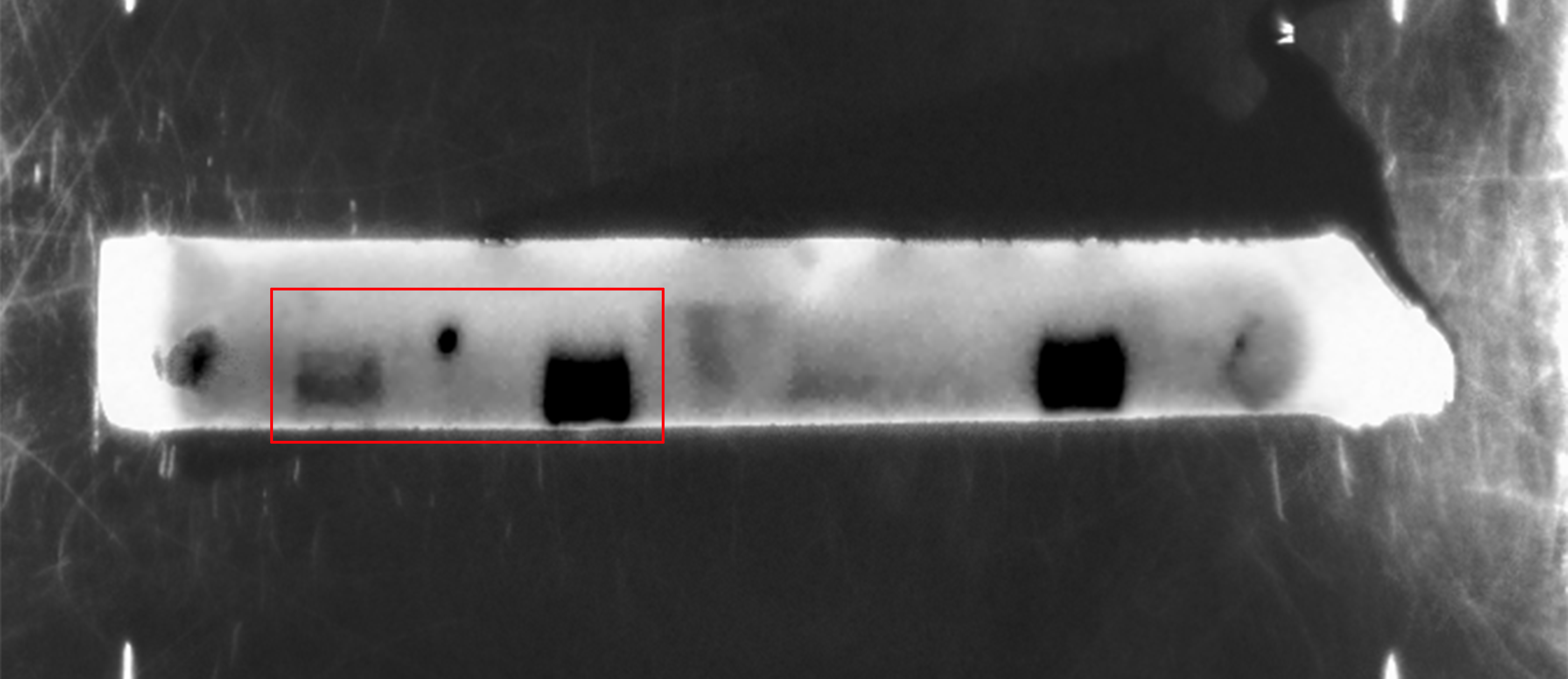


Fig. 4B Rb


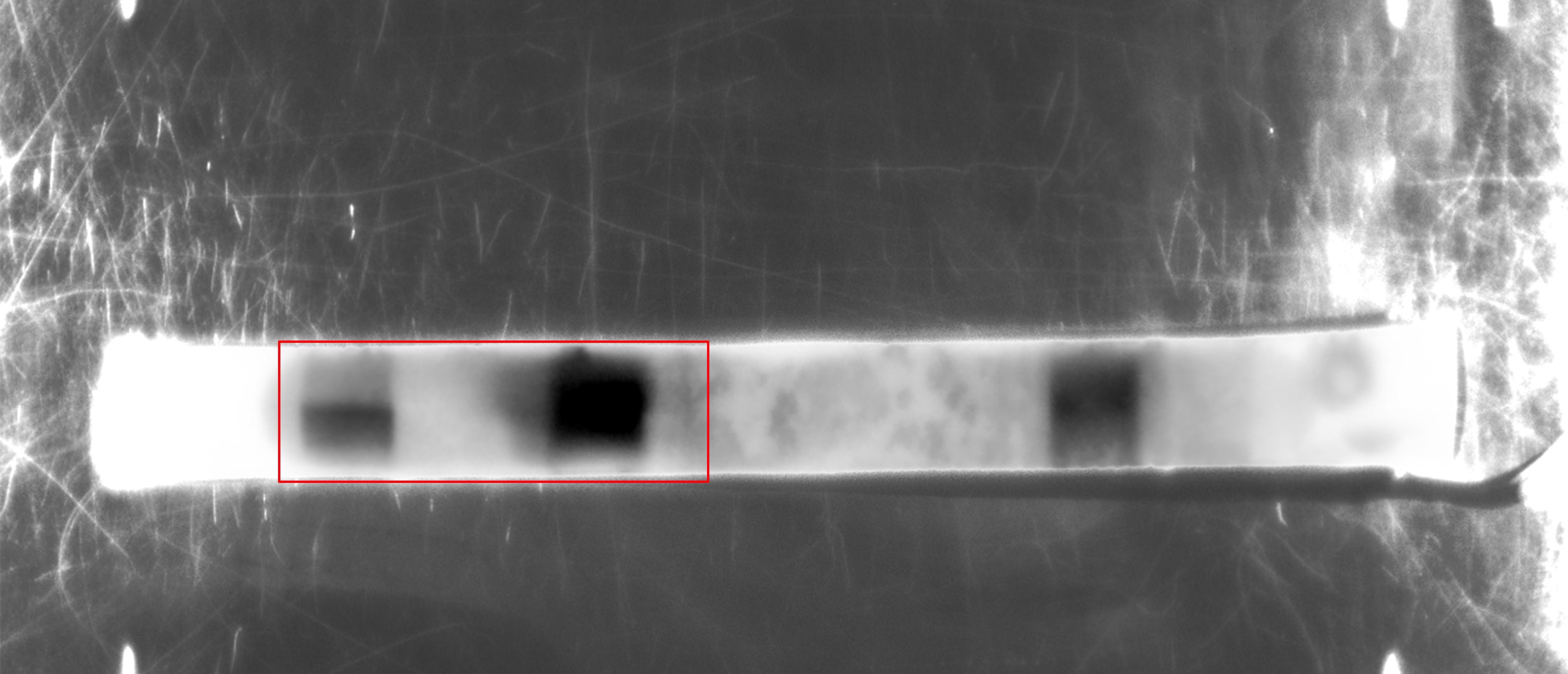


Fig. 4C CENPF


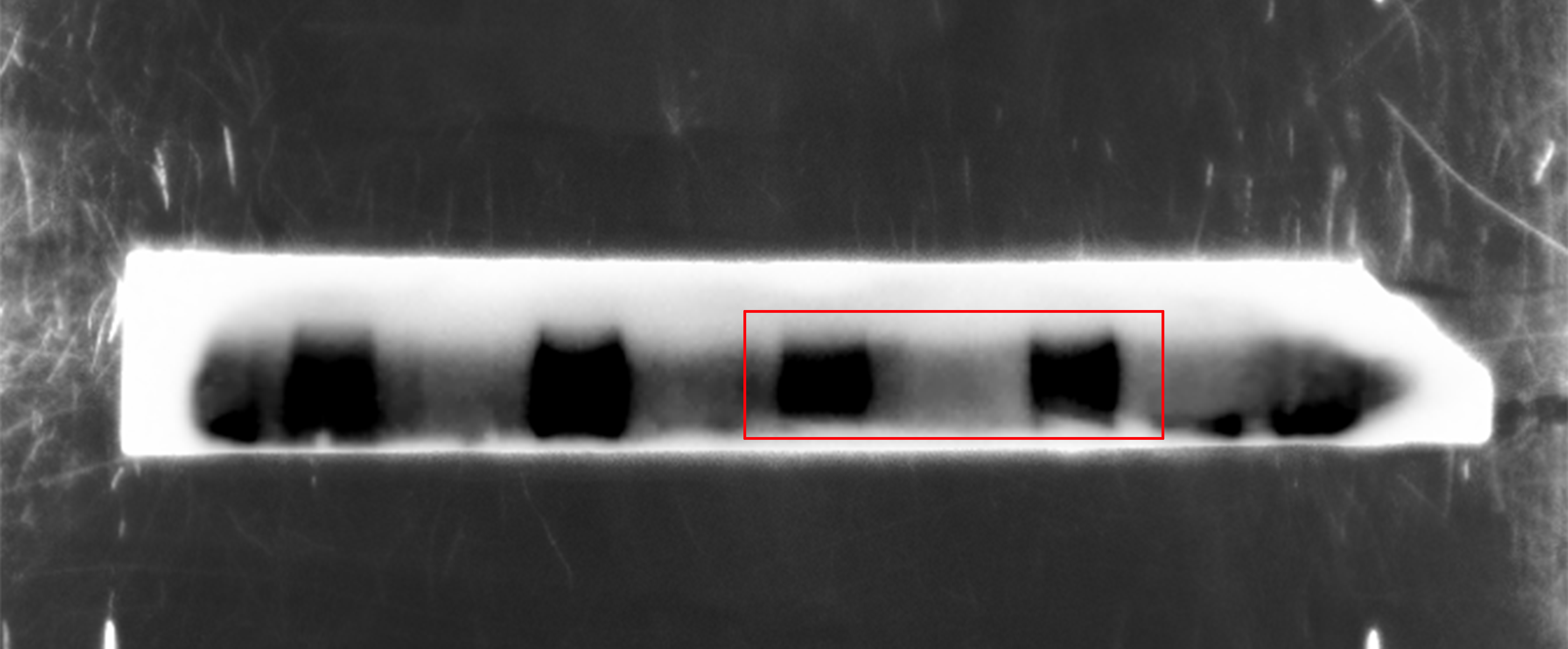


Fig. 4C Rb


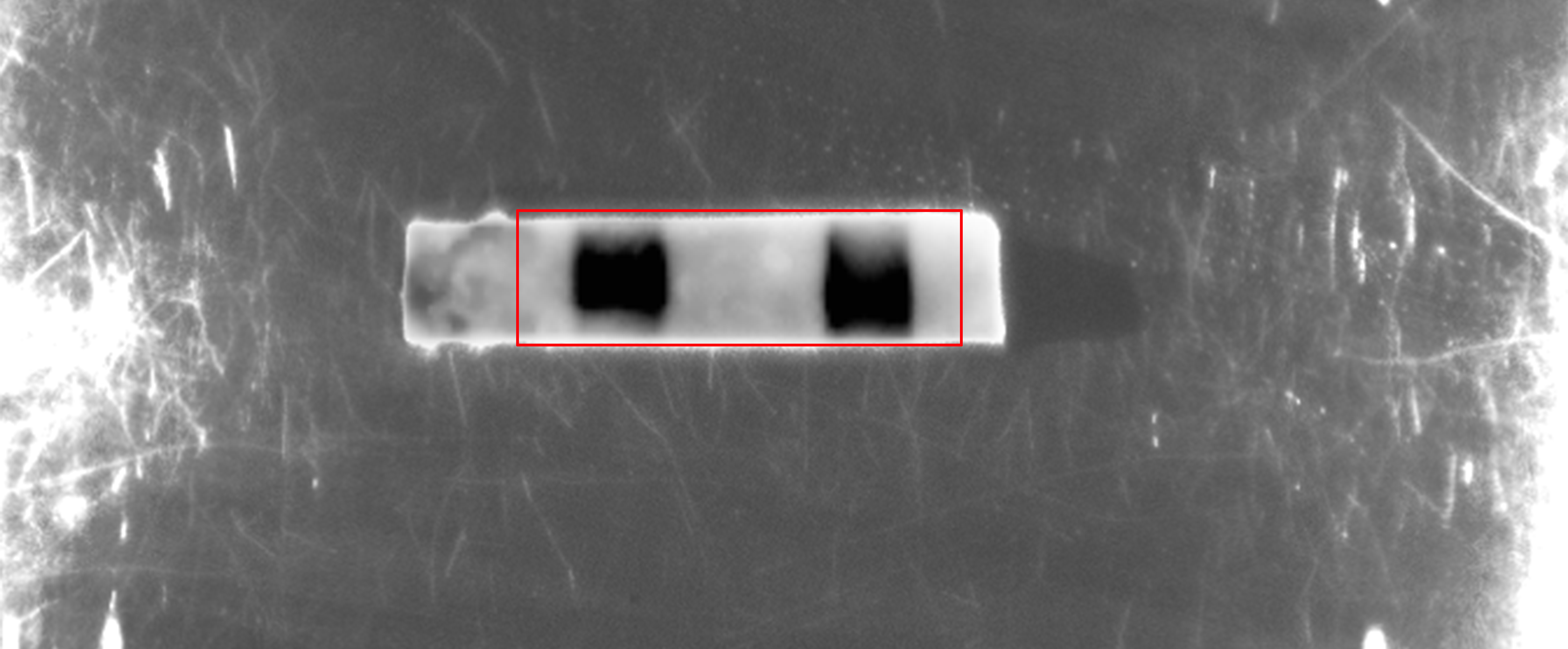


Fig. 4D Rb


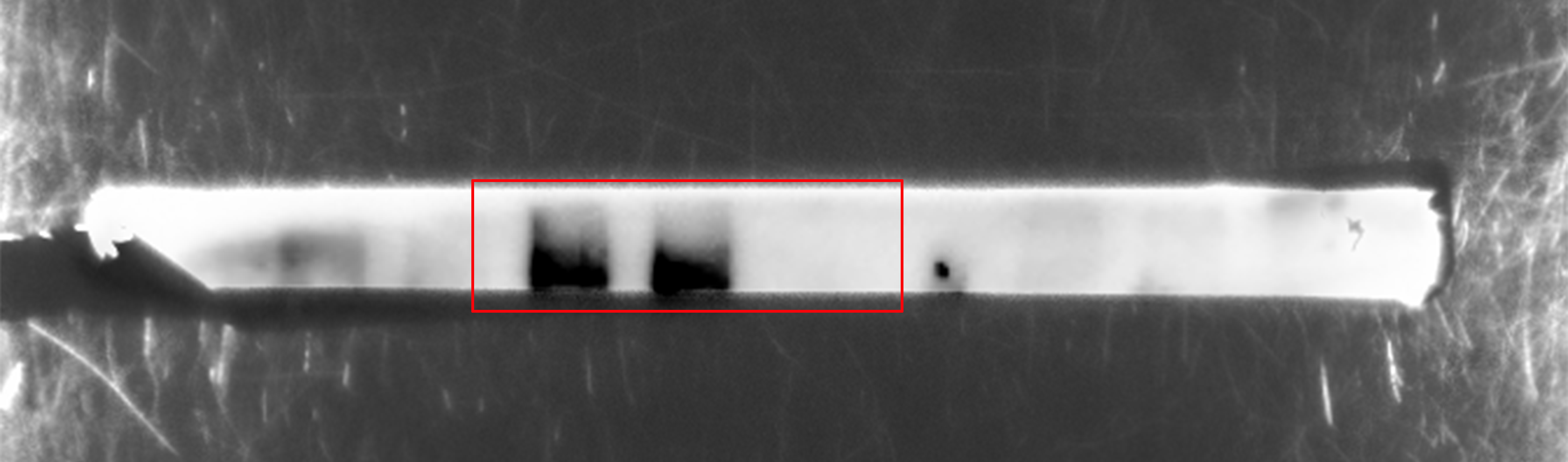


Fig. 4D E2F1


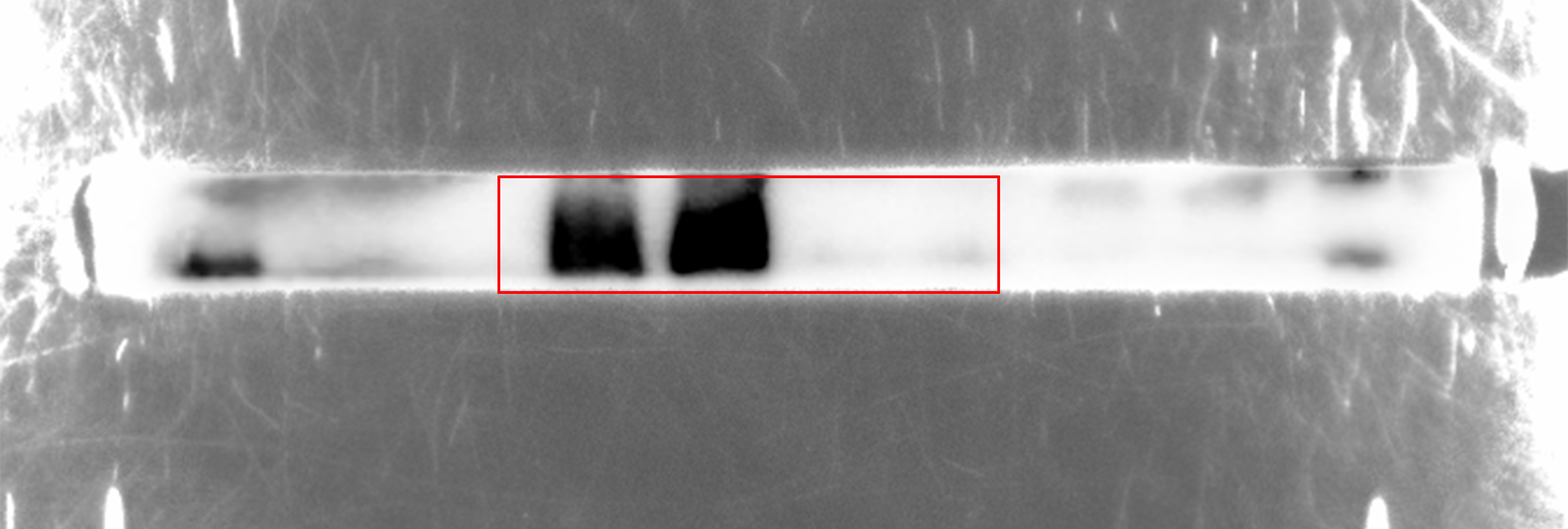


Fig. 4D CENPF


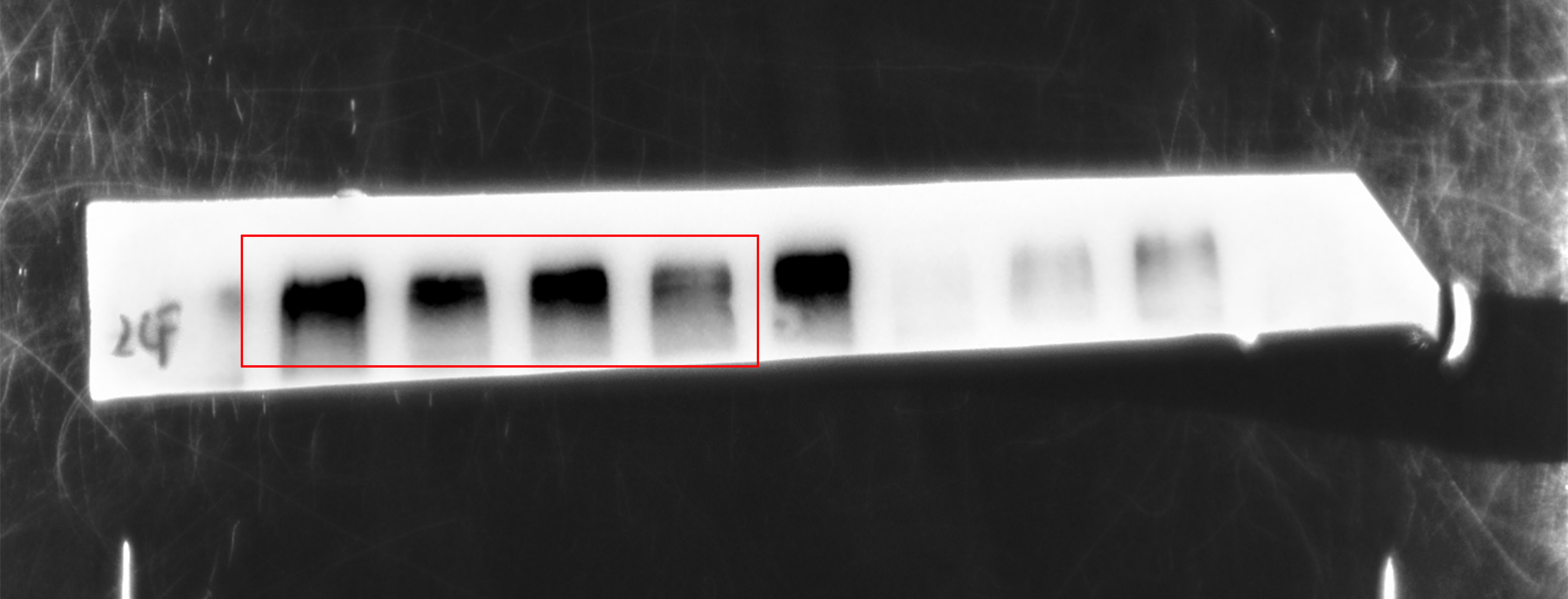


Fig. 4E CENPF


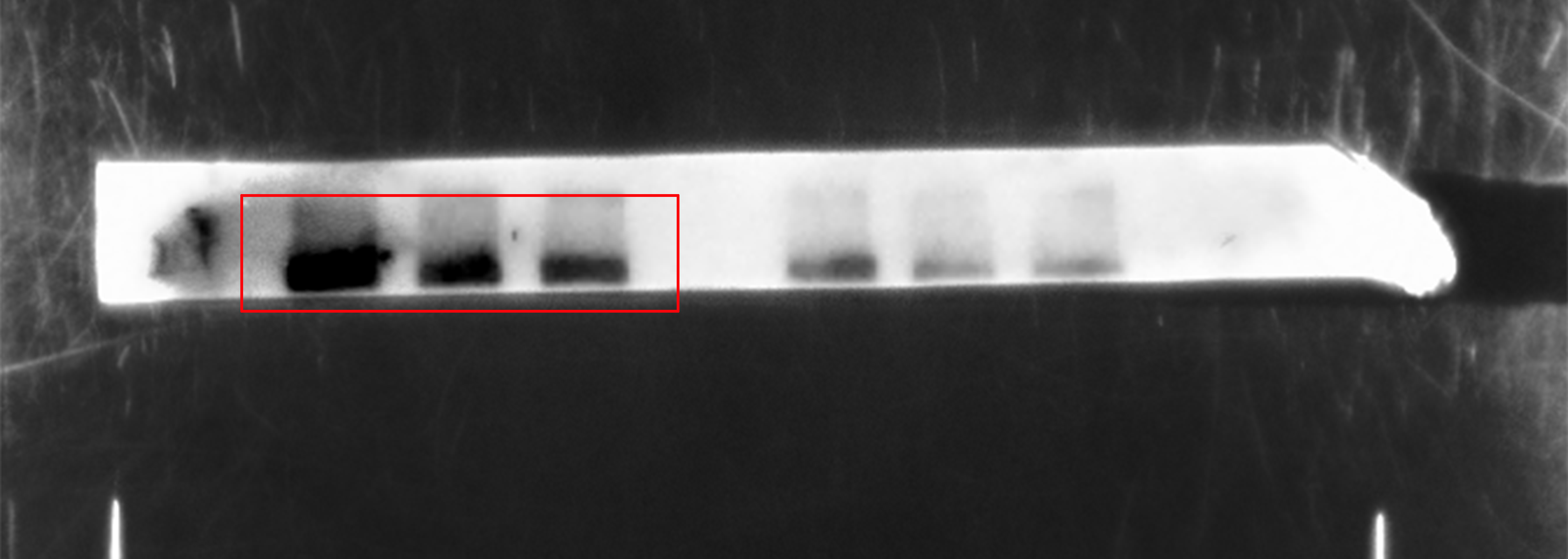


Fig. 4E E2F1


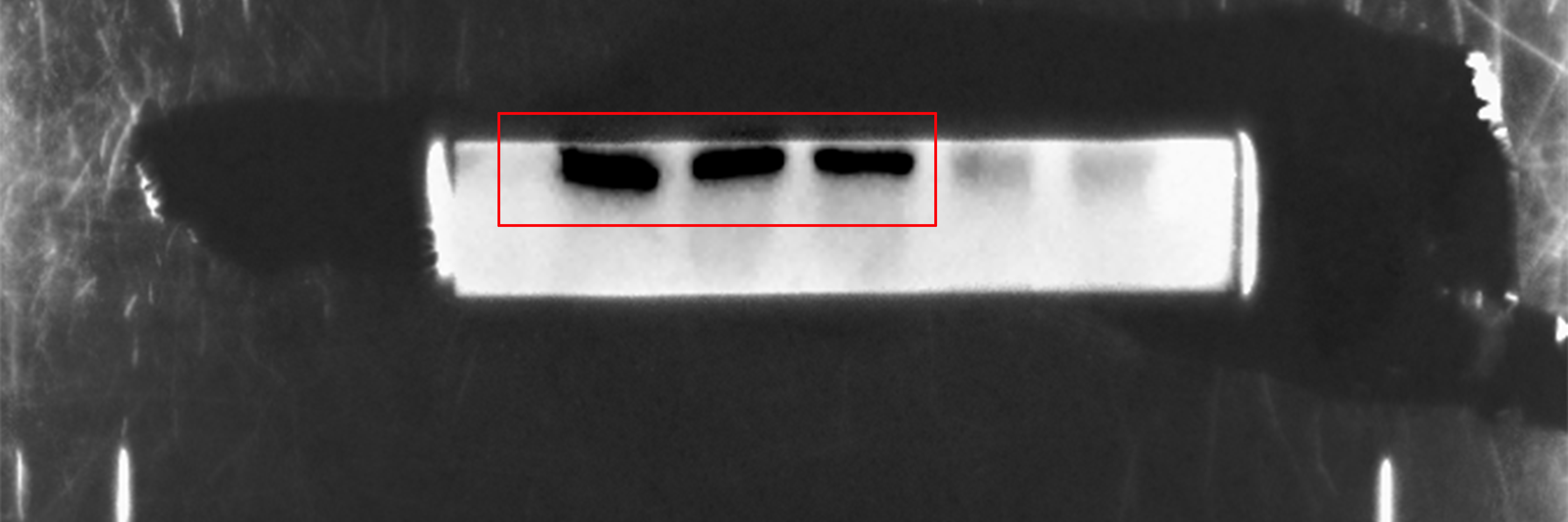


Fig. 4E GAPDH


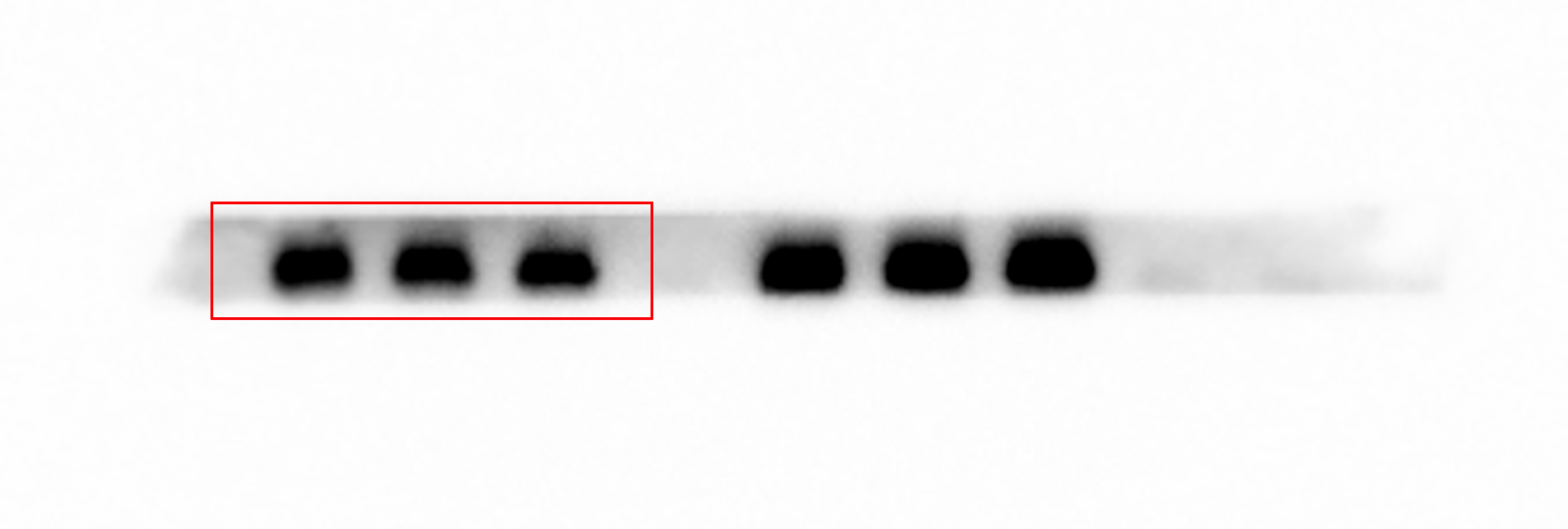


Fig. 4F CENPF


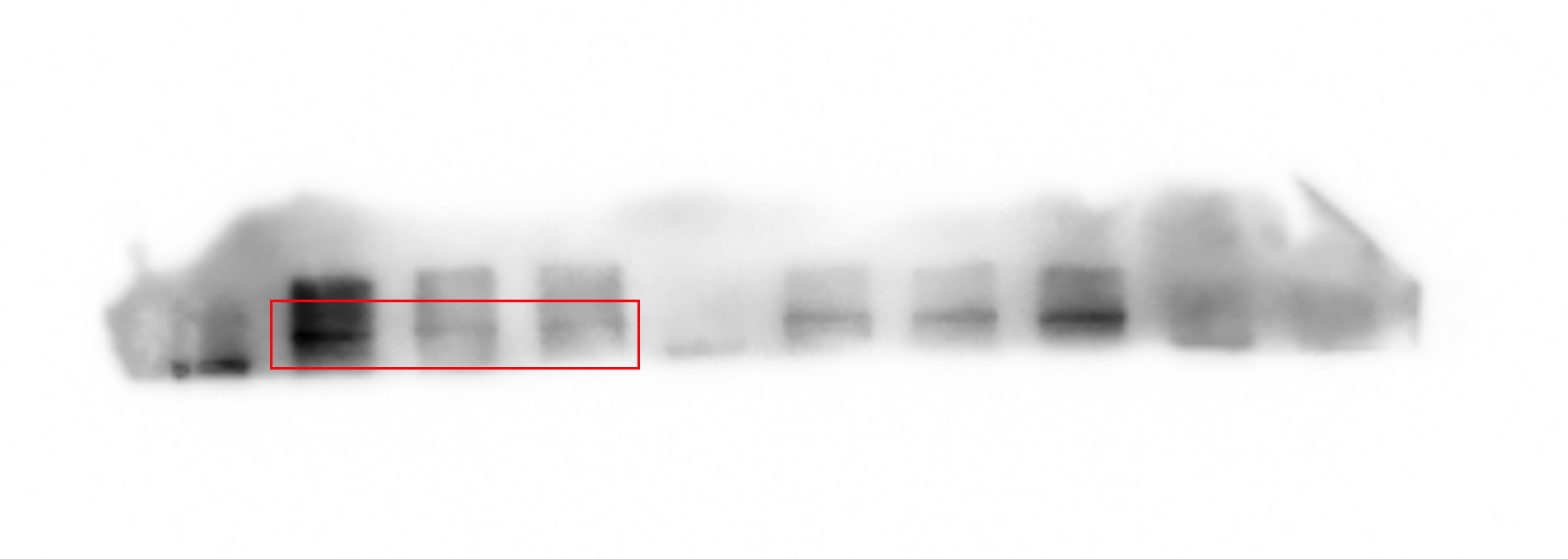


Fig. 4F E2F1


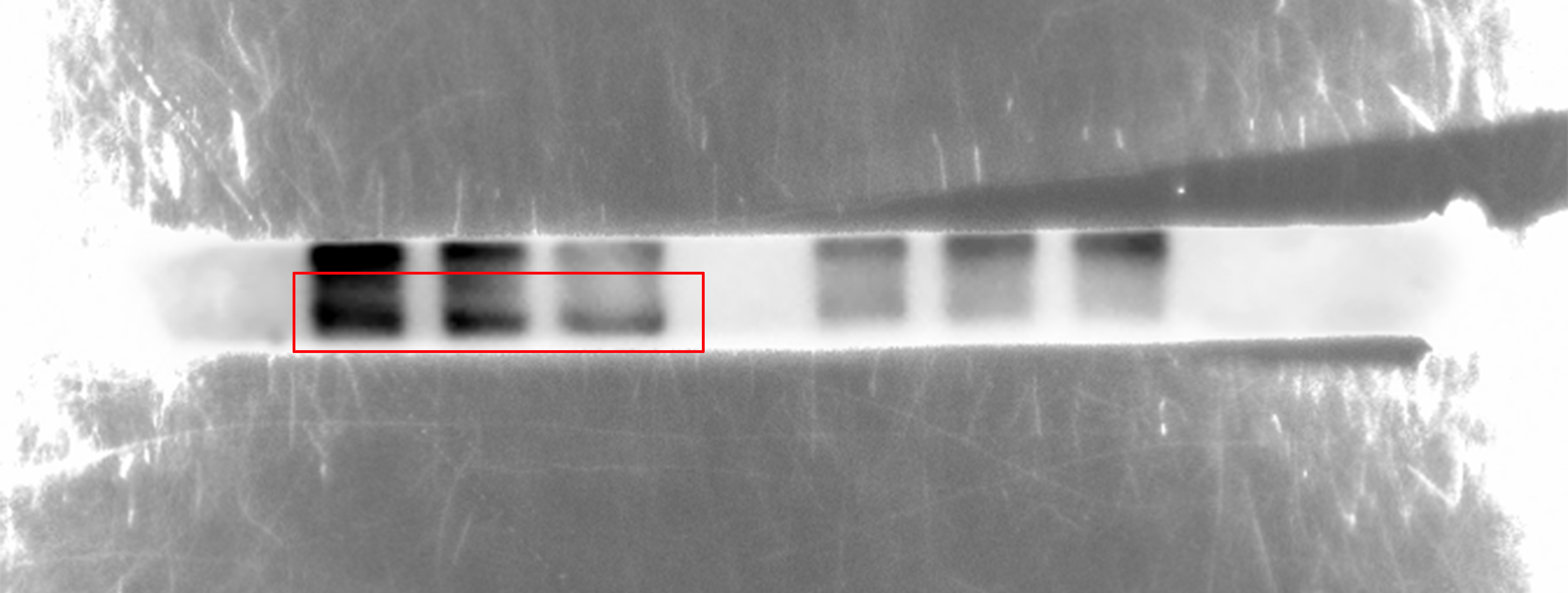


Fig. 4F GAPDH


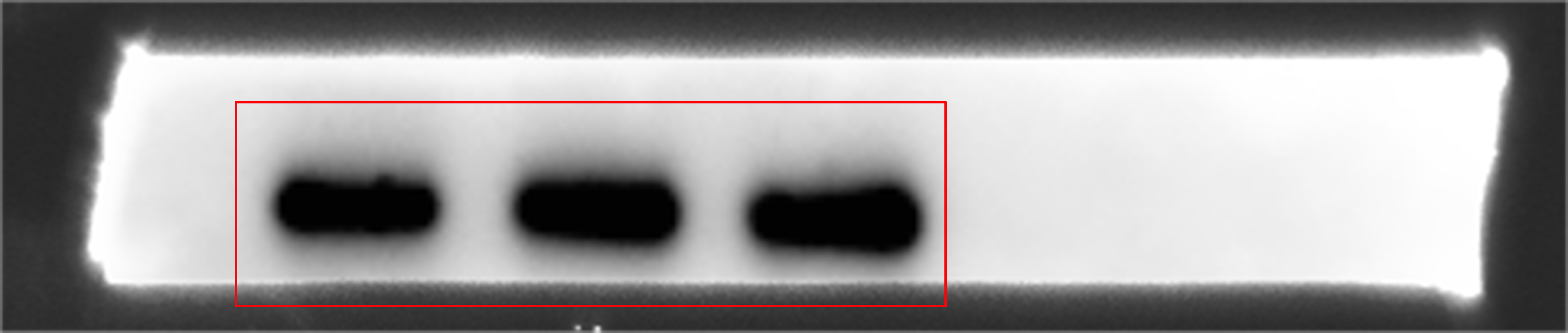


Fig. S1 CENPF


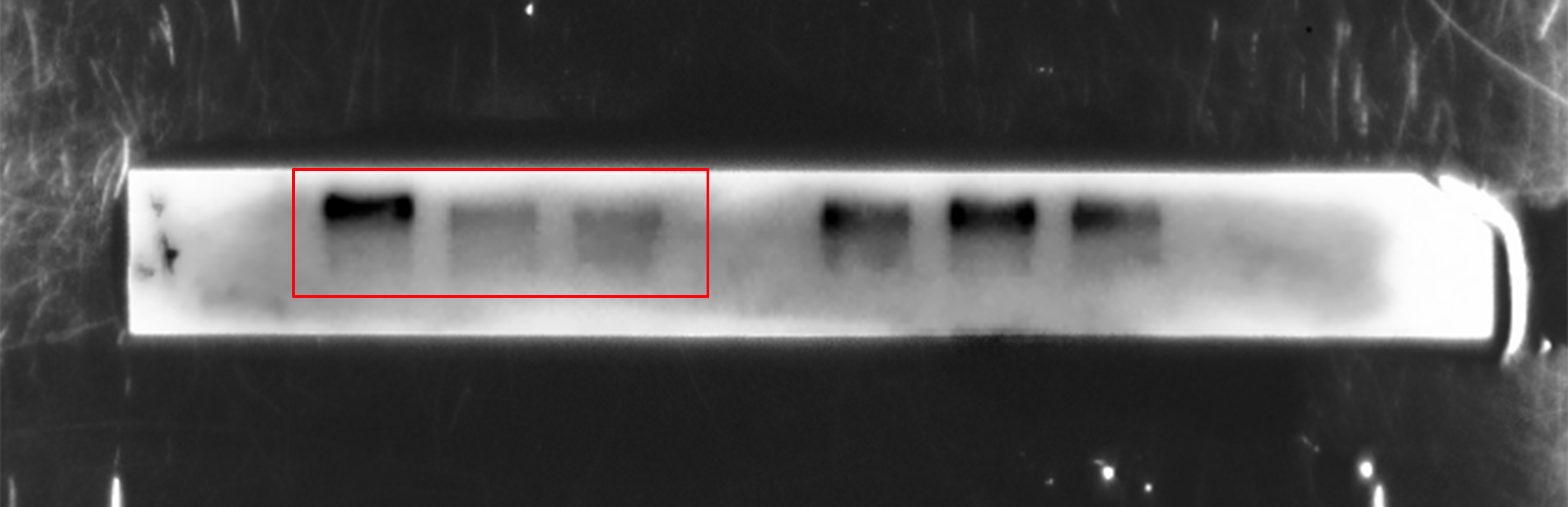


Fig. S1 GAPDH


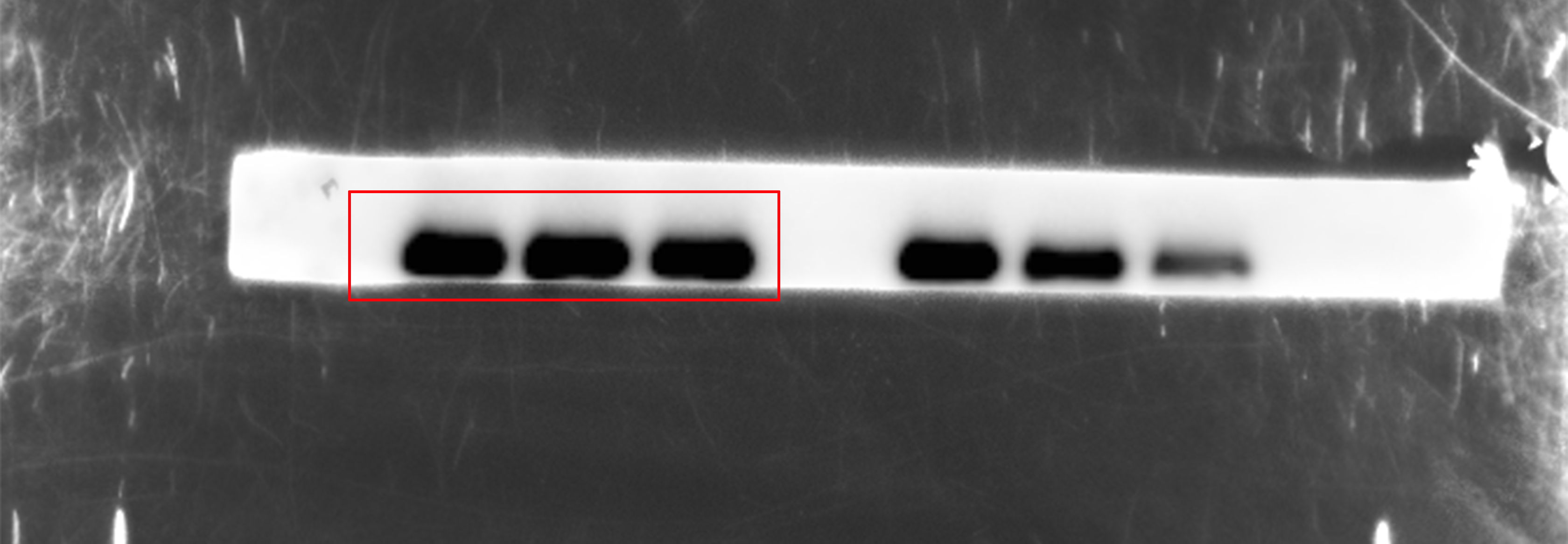


Fig. S2 CENPF


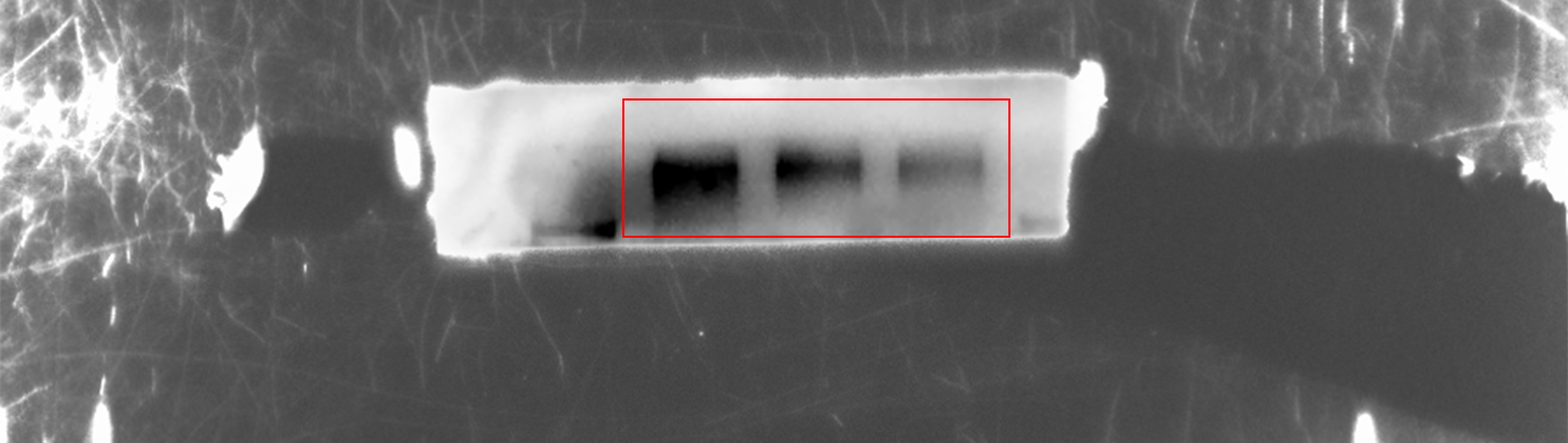


Fig. S2 GAPDH


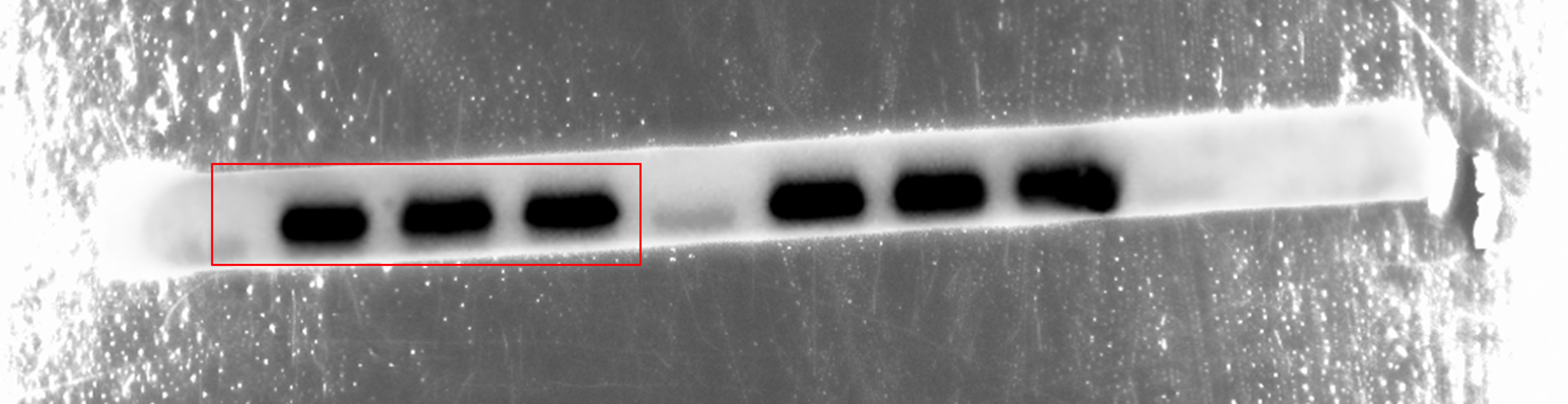

Supplement: Supplementary file 1 — Supplementary Information. [file 41598_2023_28355_MOESM1_ESM.docx]
